# Thinking you're different matters more for belonging than being different

**DOI:** 10.1038/s41598-024-58252-y

**Published:** 2024-03-30

**Authors:** Sareena Chadha, Tiffany Ha, Adrienne Wood

**Affiliations:** Department of Psychology, University of Virginia, 485 McCormick Rd, Charlottesville, VA 22904 USA

**Keywords:** Psychology, Human behaviour

## Abstract

Belonging to a community is essential for wellbeing, but potentially unattainable for those dissimilar from a group. In the present work, we ask whether belongingness is better predicted by acting and thinking like peers or *believing* you act and think like peers. Students (N = 1181) reported their belonging and how much they, their friends, and an “average student” endorsed local behavioral norms and general values. We calculated difference scores for behaviors and values capturing perceived similarity to the average, actual similarity to the average, and accuracy around the norm. Key results indicate that *perceived* behavioral similarity to the average, when controlling for other differences, predicts belonging and most robustly mediates between identity and belonging. Using social network analysis, we find behavioral differences from friends are meaningfully linked to network density and racial homophily. Efficient interventions for enhanced belonging could highlight similarities between students and their peers.

## Introduction

People want to feel that they belong^1^. Feeling connected to and accepted by a community is essential to flourishing, as belonging to a group yields access to support, resources, and a sense of meaning^2^. Students who feel they belong at their college, for instance, have lower perceived stress and greater life satisfaction^3^, more tightly-knit^4^ and supportive^5^ social networks, higher grade point averages^6^, greater college persistence, and better mental health^7,8^. Although often studied in Western contexts, belonging is equally related to increased wellbeing across several distinct nations (Belgium, China, United States, and Peru)^9^.

People who are similar to their group are likelier to report belonging^10^. Those who are marginalized economically, culturally, or racially tend to report lower belonging, partly because they are different from everyone else^11^. Indeed, daily group-level interactions that highlight identity differences (e.g., talking about travel, hobbies, or media) lead marginalized people, across numerous social identities, to experience identity threat and diminished belonging^12^. Students with a racial-ethnic minority or first-generation background report lower belonging than their peers^7,13^. Yet lack of belonging is not inevitable for people with minority identities. For instance, minoritized college students’ belongingness increases if they follow a norm of engaging in campus activities^14^. Higher belonging positively influences minority students’ academic retention outcomes^15^ and institutional commitment and persistence^16^.

Regardless of how objectively similar a person’s traits are to the “average” community member, it may matter how similar they *think* they are to the average and how accurately they assess the traits of this “average” community member. In the present work, we explore this possibility by comparing how students’ college belongingness relates to their perceived similarity, actual similarity, and accuracy regarding their peers' behaviors and values.

### Misperceptions of group behavior and values

There is reason to expect that people’s actual and perceived similarity to the group are not strongly correlated and independently predictive of important outcomes. People often misestimate the “typical” values and behaviors of their peers^17^, by overestimating (pluralistic ignorance^18,19^) or underestimating (false consensus^20^) their similarity to the group. Specifically, people make biased judgments about others’ values, compared to their own, across various groups (their family, their city, their country, or another country)^21^. When building a schema for the “average person” in a community, observers often rely on biased sampling strategies that produce inaccurate estimates^22^. They may also have biased recall, remembering some salient examples over others^23^. Nisbett & Kunda (1985) found people anchor estimates about the distribution of their peers’ behaviors and attitudes on their own^24^.

People may further misestimate norms if their identities are unaligned with the majority^25^, since sampling instances of the “average student” are distorted by similarity to that reference^23^. Research has shown that undergraduates whose sociocultural backgrounds are dissimilar from the average—those identifying as out-of-state, first-generation, low-socioeconomic status (SES), or international—misperceive norms at their schools, which may reduce their ability to thrive, belong, and be socially embedded^25–27^.

### Group-level perception stems from local social networks

Community-level behavior is often misjudged because our perceptions of the average are drawn from our relatively limited social networks^28,29^. People in a university do not and cannot see the entire student body, and instead must draw conclusions about peers based on information from close others^29^. Indeed, people have biased views on community-level values, typically anchoring those judgments on their or their family’s values^21^. Close friendships are more likely to occur between similar people, a reliable phenomenon known as homophily^30–32^. Similarities tend to covary, such that close friends are similar in many ways: close same-race friends report more shared daily activities than cross-race friends^33^. Such echo chambers might skew judgments of the “average” peer.

The effect of friends’ traits on judgments of similarity to the larger community, and thus on sense of belonging to the community, might further depend on friendship network structure. Densely interconnected networks, where friends are also friends with each other (e.g., tight cliques), more strongly reinforce behaviors^34,35^ than loosely-connected friendship networks^36,37^. If people with dense networks experience greater behavioral similarity, density may skew downstream perceptions of similarity to the wider community. Further, people with these interconnected networks report higher belonging^4^ and more social support^5^. We therefore ask whether friendship network density and homophily moderate the relationship between perceived similarity and belonging.

### Possible consequences of thinking you are different from the group

Perceptions of others’ behavior, attitudes, and values often matter more for behavior than reality^17,29,38–40^. Dyadic research suggests that perceived similarity matters over actual similarity for initial attraction and relationship intensity, among speed daters^41^ and same-sex friends^31,42^. Much this work^8^ focuses on (a) singular targets and (b) similarity in personality or attitudes predicting attraction. The present study extends this framework (a) by instead investigating perceived similarity to a general hypothetical peer and one’s friendship network and (b) from trait-based similarity to similarity in social behaviors (which are more mutable) and values predicting belonging (a novel consequence from perceived and/or actual similarity). Belonging is subjective and relies on individuals’ construal of their social context^43^—thinking you hold values that differ from an ingroup negatively impacts belonging^26^. Simply holding values that conflict with ingroup members’ behavior reduces group identification^44^. In this study, we ask if holding values (or endorsing behaviors) that conflict with perceived or actual values (or behaviors) of the community matter. We hypothesize that perceived similarity to a community will matter more for sense of belonging than actual similarity on that same dimension.

Limited work has looked at this perceived similarity-belonging link directly. Most work focuses on a single behavior, such attending an eating club at an Ivy League school^45^ or using well-known university spaces^46^. One study found that college students who believed they were different from their peers identified less with the university^26^. This effect was moderated by SES, such that low-SES students perceived a greater difference between their norms and values of the average student. However, this work only assessed self-reported norm and value differences between the self and the average student using a single survey item: “In general, how much do you think your norms and values are different from the average student?”^26^. This research did not control for actual similarity to the average student, so the effect could be driven by students’ accurate perceptions of their own dissimilarity. From this work, we do not know whether the effect is driven by differences in concrete and observable behavior or abstract values. Some work has emphasized the effects of self-other norm^25^ and value^47^ differences separately on wellbeing; to design effective interventions, we must unpack which variables strongly and consistently predict belonging, given that behavior change is difficult^48^.

### The present work

We address this gap by conducting a correlational study at a U.S. university, which is a preregistered replication and extension of a preregistered pilot study (see Supplementary Materials for pilot study results; OSF page https://osf.io/wv5r6/?view_only=b812a856a3944cc190e556ac0678501d).

Participants reported their engagement with specific behaviors and their endorsement of abstract values, and how much they believe the “average student” engages in those same behaviors and holds those values. We calculated three scores separately for behavior and values: (1) students’ *perceived* similarity to their understanding of the average student (self-average similarity), (2) their *actual* similarity to the sample mean (self-sample similarity), and (3) their perception of the norm compared to the sample mean (our proxy for the “true” norm; average-sample similarity). Each calculation captures one dimension of being different from one’s peers, whether perceiving oneself as different, truly being different, or having a poor estimate of the norm.

We hypothesized that students whose behavior and values were closer to their perception of an average student’s normative behaviors and values would have a greater sense of belongingness, over their actual similarity to and accuracy about the sample average. We also hypothesized that students identifying with a minority background would exhibit lower belonging and this association would be mediated by their self-average difference.

Participants additionally reported their perceptions of their friends’ behaviors and values to determine whether perceived similarity to friends predicted belonging independently of similarity to the larger student population. We proposed that students whose behavior and values are closer to their perception of their friends’ would have a greater sense of belongingness, regardless of their perceived similarity to the wider community. Students’ perception of norms hinge on their close networks^28^; it is therefore possible that college students think of their close friends when estimating the behavior or values of the average peer^49^.

We also hypothesized that students with networks characterized by high racial homophily would perceive those friends to be more similar to them in terms of behavior and values. We reasoned that having strong ties and/or a salient shared social identity (race), should lead students to assume their friends share other commonalities. Finally, we hypothesized that the effect of perceived self-friends similarity on belonging would be greater for denser friendship networks. We reasoned that having more interconnected friends should foster feelings of community and belonging, but only when those friends are perceived as similar to the self.

In sum, the present research uses a novel approach to quantifying actual and perceived similarity based on specific behaviors and values, rather than global self-reported perceived similarity. To examine the importance of similarity observability for belonging, we quantify similarity at two levels of concreteness: for everyday behavioral tendencies and abstract values. We explore how belongingness relates to perceived similarity to friends—and how the structure of the network connecting those friends moderates the effect. This work uses novel methods to demonstrate a classic social psychological idea: the importance of social world construal^43^.

## Results

### Differences in behavior and values predict belongingness

**Figure 1:**
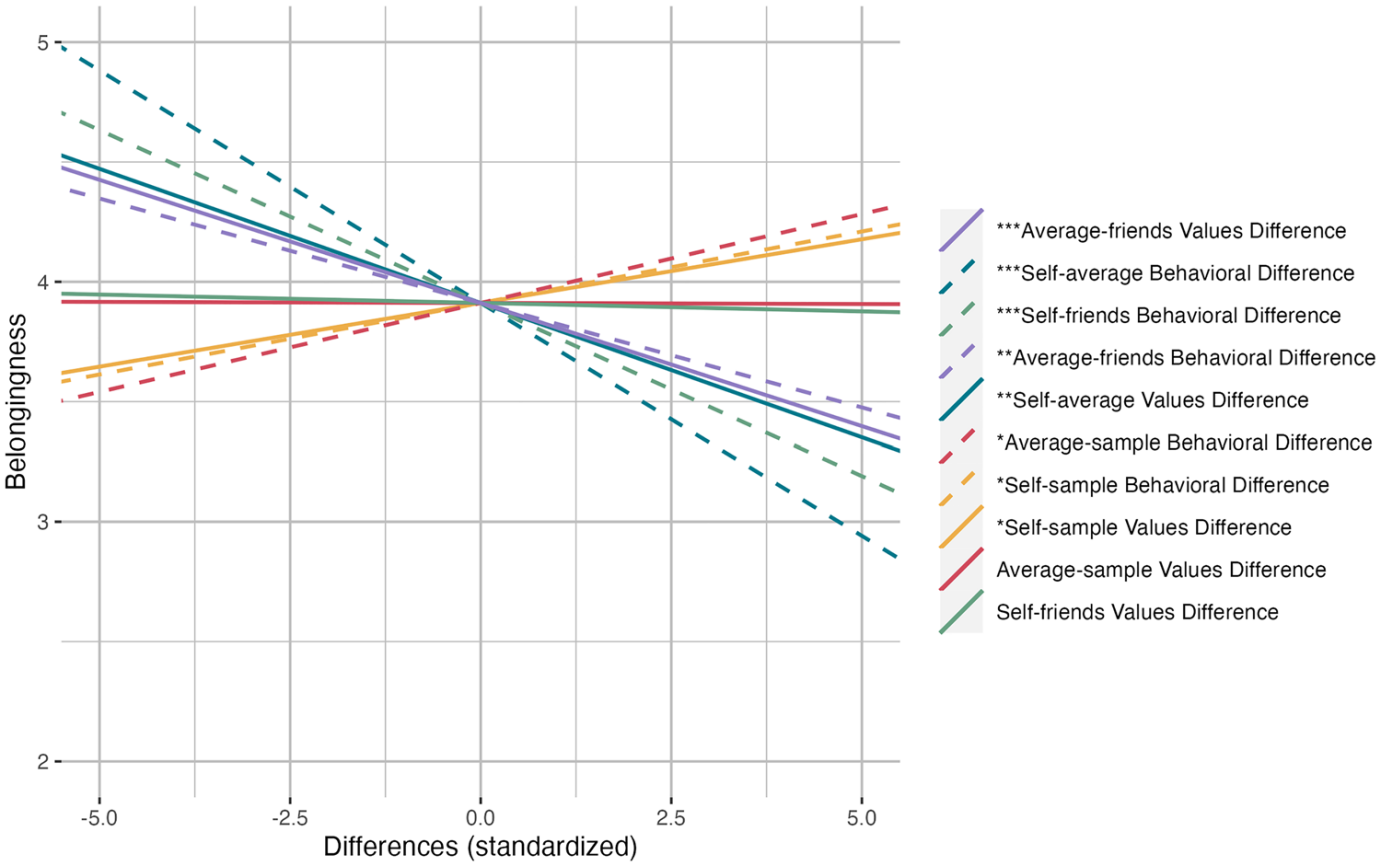
Model predictions from a regression predicting belongingness using behavior (local and general combined) and values differences scores (standardized). Dashed lines represent behavioral differences, while solid lines represent differences in values. (***p < .001, **p < .01, *p < .05).

We specified a model with the 10 difference scores predicting belongingness (Fig. 1). *Self-average behavioral difference* (*b* = − 0.194, *SE* = 0.043, *t*(1155) =  − 4.527, *p* < 0.001, η^2^ = 0.017, CI = [− 0.278, − 0.11]) and *self-average values difference* (*b* = − 0.112, *SE* = 0.035, *t*(1155) =  − 3.235, *p* = 0.001, η^2^ = 0.009, CI = [− 0.18, − 0.044]) negatively predicted belongingness, such that students who rated themselves very differently from their perception of the average student’s behavior *or* values felt as though they belong less at their university. Unexpectedly, we found that, controlling for perceived differences, students who engaged in different behaviors or endorsed values differently from the sample mean report *higher* levels of belongingness (*self-sample behavioral difference: b* = 0.06, *SE* = 0.026, *t*(1155) = 2.296, *p* = 0.022, η^2^ = 0.005, CI = [0.009, 0.11]; *self-sample values difference*: *b* = 0.053, *SE* = 0.025, *t*(1155) = 2.092, *p* = 0.037, η^2^ = 0.004, CI = [0.003, 0.103]). Note, however, that there is a weak *negative* bivariate correlation between belonging (Fig. 8) and these *self-sample difference* variables, which likely reversed in the multiple regression because self-sample differences are correlated with self-average differences (Fig. 8). This pattern may reflect a suppression effect. When we remove the portion of perceived differences that is due to a student actually *being* different, all that remains is the students’ biased beliefs about themselves and their peers, which then has an even stronger association with sense of belonging. Having a biased view of how different one is from peers is the strongest predictor of sense of belonging, over self-sample behavioral and values differences.

Students who rated their perception of an average student’s behavior differently from the sample mean behavior *(average-sample behavioral difference)* reported greater belonging (*b* = 0.074, *SE* = 0.031, *t*(1155) = 2.361, *p* = 0.018, η^2^ = 0.005, CI = [0.013, 0.136]). As with the *self-sample difference* variables, *average-sample behavioral difference* seems to be playing a suppressor role. *Average-sample values difference* was unrelated to belonging in this model. Students who rated their friends’ behaviors differently from their own (*self-friends behavioral difference)* were inclined to feel lower levels of belongingness (*b* = − 0.144, *SE* = 0.029, *t*(1155) =  − 4.971, *p* < 0.001, η^2^ = 0.021, CI = [− 0.061, − 0.047]); this pattern did not replicate for *self-friends values difference*. Students reported lower belonging if they rated their friends’ behaviors or values very differently from their perception of an average student’s behaviors (*average-friends behavioral difference; b* = − 0.087, *SE* = 0.032, *t*(1155) =  − 2.72, *p* = 0.006, η^2^ = 0.006, CI = [− 0.15, − 0.024]) or values (*average-friends values difference; b* = − 0.103, *SE* = 0.03, *t*(1155) =  − 3.425, *p* < 0.001, η^2^ = 0.01, CI = [− 0.162, − 0.044]).

In sum, the largest effects on belonging were for self-average behavioral difference and self-friends behavioral difference: *perceiving* yourself to be similar to your peers or friends predicts belonging, regardless of how similar you actually are. Supplementary exploratory analyses suggest that believing oneself to be similar to peers in *location-specific* behaviors matters more for belonging than believing oneself to engage in similar *generic* behaviors.

### Differences in behavior mediate social identity’s effects on belongingness

**Figure 2:**
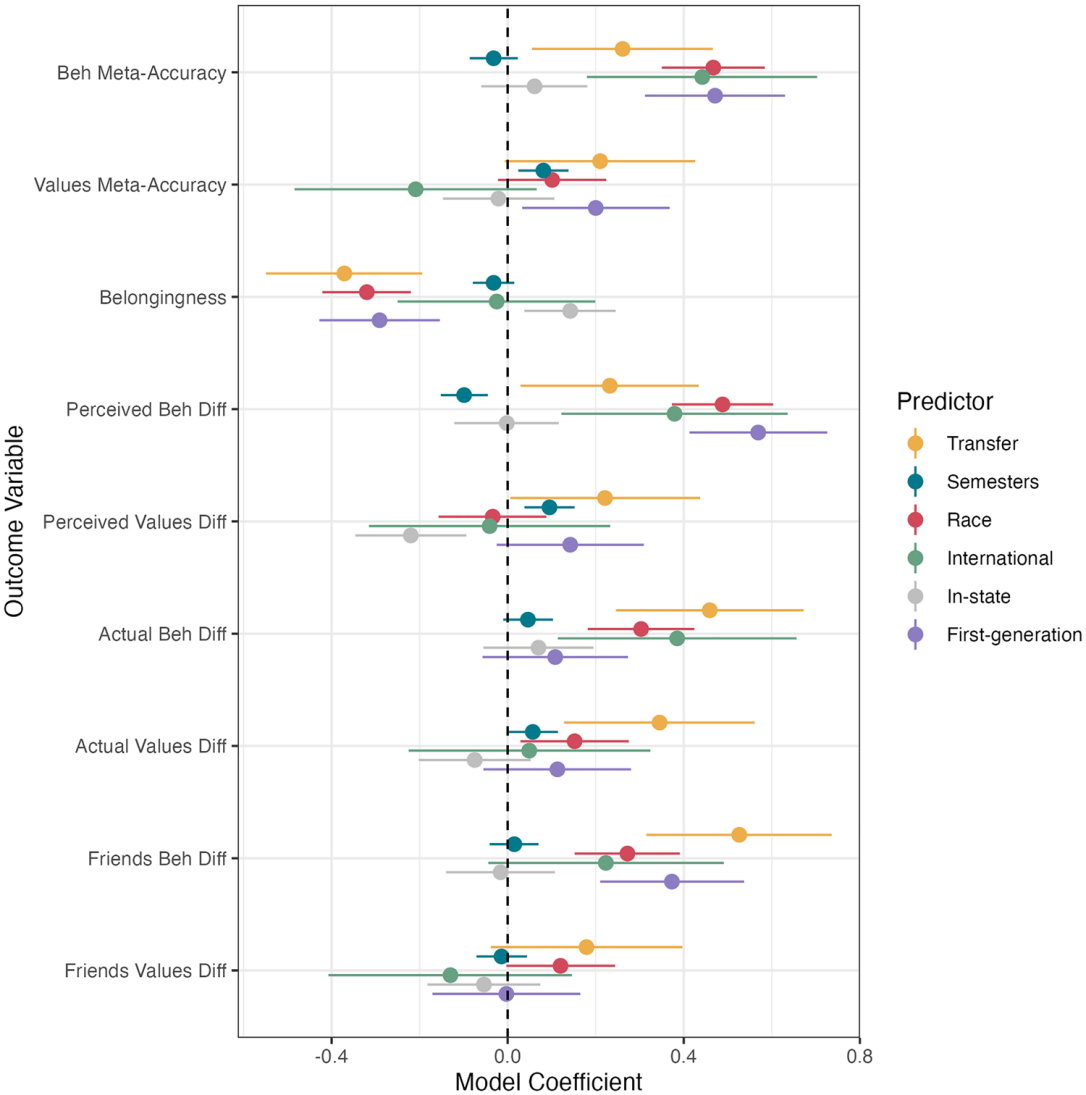
Beta coefficients for semesters spent on campus and social identity facets on belongingness and difference scores. Difference scores were analyzed if they were included in subsequent preregistered mediation models in Fig. 3 (See OSF for average-friends differences). Significant relationships exclude 0. For numerical beta coefficients and p-values, see Supplementary Materials, Table 5.

We know that perceived and actual differences relate to belongingness (Fig. 1), which led us to ask if these differences could mediate the relationship between various dimensions of social identity and belongingness. Before running our preregistered mediation models, we first identified significant relationships between social identity variables and (a) belonging and (b) behavior and value differences to avoid errors in assuming indirect effect significance without significant direct effects^50^, which was not preregistered. After this step (Fig. 2; details reported in Supplementary Materials), we tested for mediation with race, in-state, first generation, and transfer student status, all of which predicted belonging (Fig. 2).

**Figure 3:**
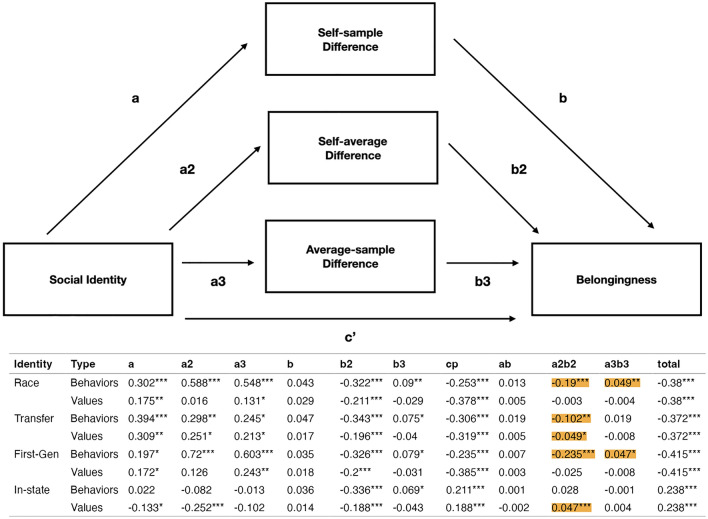
We ran identical multi-path mediation models for each of the social identities listed in the table, first with behavior-based difference scores as mediators, then with values-based difference scores as mediators. Highlighted are the significant indirect effects of self-average differences (whether behavior or values; a2b2 path) and average-sample differences that partially mediated the relationship between social identity and belongingness when controlling for all other differences (***p < .001, **p < .01, *p < .05).

We specified structural equation models with (a) a direct path from social identity to belongingness, (b) indirect simultaneous paths from social identity to belongingness via self-average, self-sample, and average-sample behavioral or value differences in separate models, and (c) covariances between difference scores (Fig. 3; See OSF for details). We also estimated the statistical significance of (d) the three indirect effects, (e) the total effect (direct and indirect effects combined) and (f) the contrasts between the three indirect effects, which indicated whether one mediator was a significantly stronger mediator than another.

We first examined if behavioral differences mediated the relationship between racial identity and belongingness. The negative indirect effect (a2*b2 path in Fig. 3) of race (with students of color coded as 0.5 and White coded as − 0.5) on belongingness via *self-average behavioral difference* was significant (*b* = − 0.19, *SE* = 0.028, *p* =  < 0.001, *δ* = − 0.19, *CI* = [− 0.244, − 0.136]), while controlling for self-sample and average-sample behavioral differences. The data are compatible with a model in which students of color feel like they belong less, in part, because they perceive their everyday behavior to be different from their peers’. The positive indirect effect of race on belongingness via *average-sample behavioral difference* was significant (*b* = 0.049, *SE* = 0.018, *p* = 0.006, *δ* = 0.049, *CI* = [0.013, 0.085]), while controlling for self-sample and self-average behavioral differences (a3*b3 path in Fig. 3). Controlling for the other effects, students of color tended to have less accurate estimates of the “average” student’s behavior, but this actually predicted *greater* belonging. We interpret this as a suppression effect, since exploratory models with just average-sample difference as a mediator follow the same direction as the self-average difference effects (See OSF). In other words, when we control for the indirect effects of student race on belonging via perceived and actual differences from peers, it uncovers a buffering effect of misjudging the true behavior of their peers.

The contrasts comparing the indirect effects via *self-average* and *self-sample behavioral differences* (*b* = − 0.203, *SE* = 0.031, *p* < 0.001, *δ* = − 0.203, *CI* = [− 0.264, − 0.141]) and *self-average* and *average-sample behavioral differences* (*b* = − 0.239, *SE* = 0.041, *p* < 0.001, *δ* = − 0.239, *CI* = [− 0.319, − 0.159]) were significant. Perceiving oneself to be different from peers is a significantly stronger mediator between race and belonging than actual difference or having accurate perceptions of the norms.

This pattern replicated for first-generation students—their greater *average-sample behavioral difference* led to greater belonging and greater *self-average behavioral difference* led to less belonging (Fig. 3). Additionally, we found evidence that fit with a mediation model for transfer status (Fig. 3), such that transfer students exhibited greater self-average behavioral differences and thus lower levels of belongingness. This pattern did not replicate for out-of-state students.

Furthermore, *self-average values difference* partially mediated the relationships between transfer student status and in-state status on belongingness while controlling for self-sample and average-sample differences (Fig. 3). This pattern was insignificant for racial identity and first-generation student status.

To summarize thus far, perceiving one’s behaviors and values far from the average student’s predicted reduced belonging to the university community. Perceiving oneself to engage in different behaviors than peers emerged as a consistent mediator of the effect of various marginalized social identities on belonging. Generally, these preregistered analyses replicated our pilot study. Next, we examined how perceived difference from friends interacts with the structure of those friendships to predict belongingness.

We preregistered mediation analyses that paralleled those presented in Fig. 3, asking if self-friends differences in either behavior or values might partially mediate social identity effects on belongingness, and found this was true for students of color, those who transferred, or were first-generation (See Supplementary Materials, Fig. 4). Once again, the data are consistent with a model in which minoritized students feel like they belong less, partly because they believe themselves to behave differently from their peers—this time, from their friends.

### Friendship network density moderates the relationship between behavioral differences and belongingness

We demonstrated that perceiving oneself to be different from one’s friends is associated with reduced belonging. We believed this effect might be exacerbated if those friends are tightly-knit. It is presumably marginalizing to feel different from a cohesive group of people who have many things in common with each other (as groups tend to)^51^.

**Figure 4:**
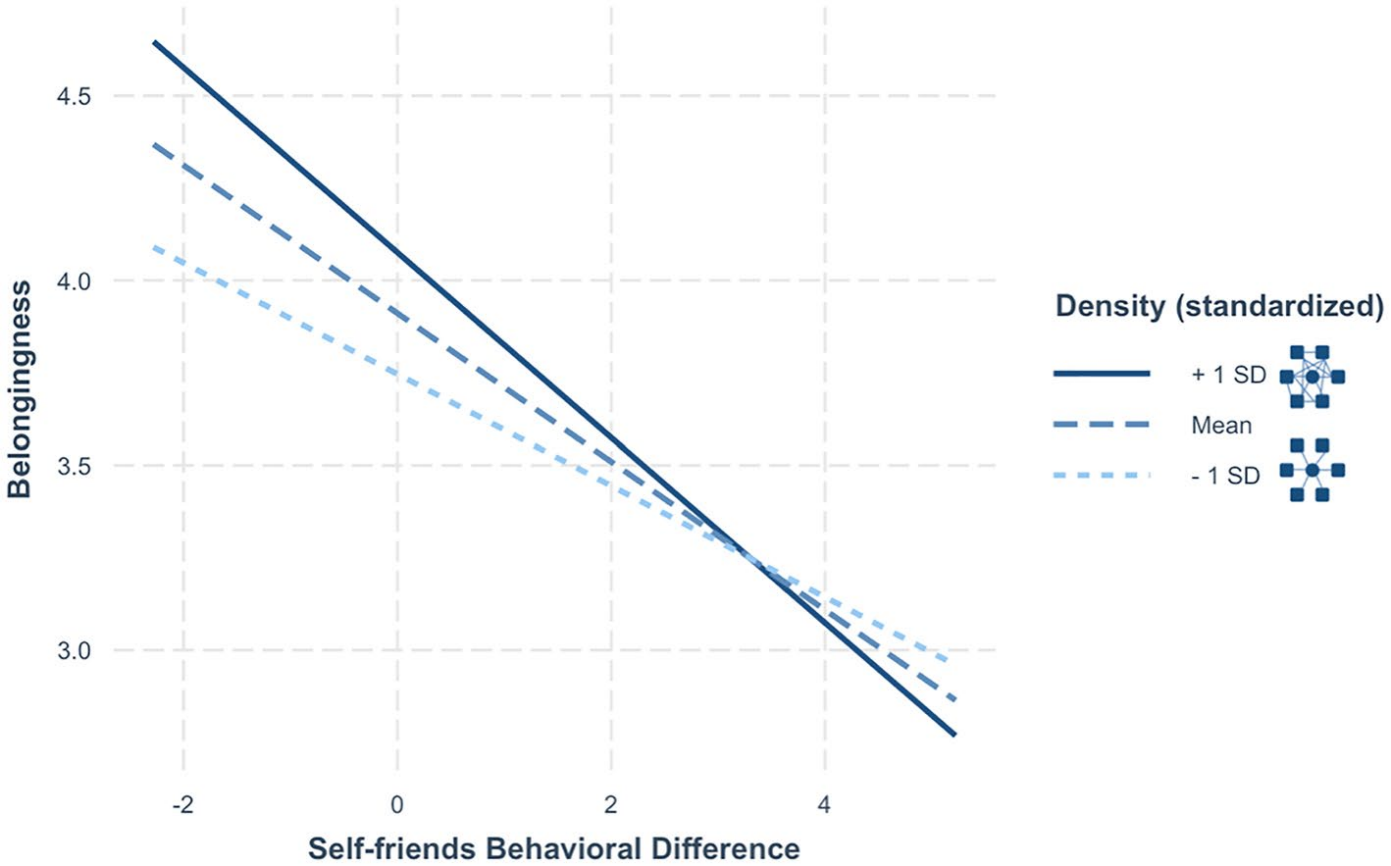
Model predictions of the relationship between perceived behavioral difference from friends and belongingness, significantly moderated by network density.

To test this, we specified a preregistered model with *self-friends behavioral difference* and mean-centered density interacting to predict college belonging (Fig. 4). Students who reported friendship networks where most their friends knew one another (higher density) reported greater belongingness (*b* = 0.165, *SE* = 0.024, *t*(1144) = 6.997, *p* < 0.001 η^2^ = 0.039, CI = [0.118, 0.21]). If students rated their behavior far from how they rated their friends’ (*self-friends behavioral difference)*, they reported lower belonging (*b* = − 0.2, *SE* = 0.024, *t*(1144) =  − 8.2, *p* < 0.001 η^2^ = 0.053, CI = [− 0.248, − 0.153]), consistent with previous results. This relationship, as predicted, was moderated by density (*b* = -0.05, *SE* = 0.023, *t*(1144) =  − 2.198, *p* = 0.028 η^2^ = 0.004, CI = [− 0.094, − 0.005]). Students with dense networks reported higher belonging, but this relationship diminished if they rated their friends’ behaviors differently from their own. We ran this same model with *self-friends values difference* interacting with density and only found a significant main effect of density on belonging (See OSF).

We specified exploratory mediation models with self-friends difference mediating between network density and belonging. We found a significant indirect effect for behavioral (not value) differences, such that those with tight-knit networks rated their behavior close to their friends’, and in turn reported lower belonging (See Supplementary Materials, Fig. 5). Additional preregistered analyses in Supplementary Materials showed that people with denser friendship networks generally perceived their behavior to be more similar to the average student.

### Having mostly same-race friends predicts perceived similarity to friends for white students but not students of color

**Figure 5:**
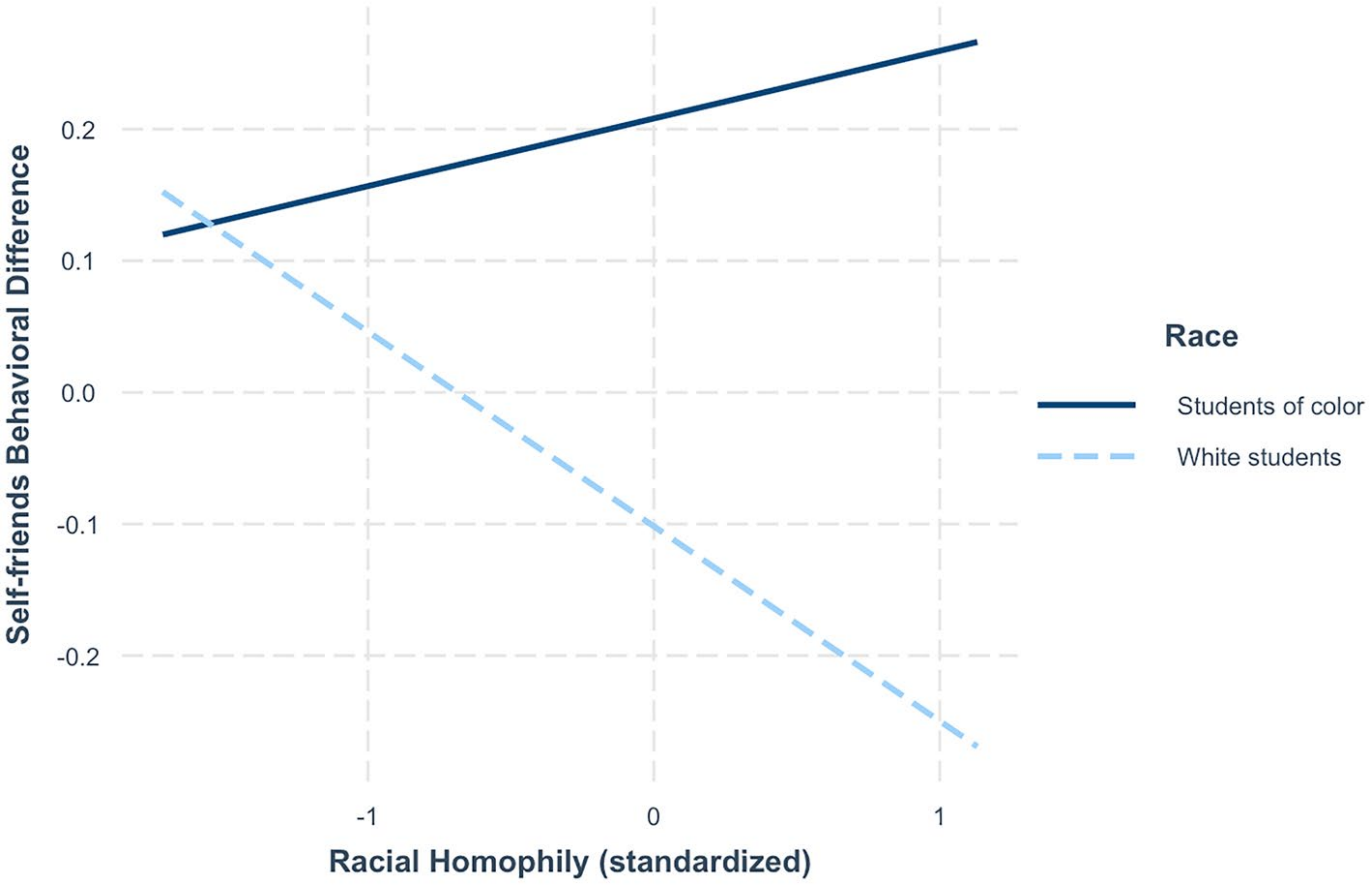
Model predictions of the relationship between racial homophily and self-friends behavioral difference (how similar students perceive their own behavior and the behavior of their friends to be), significantly moderated by racial identity.

We predicted that greater racial homophily, or a student’s proportion of friendships that are same-race, would negatively predict perceived differences from those friends, and that this effect will be moderated by students’ own racial identity. We specified a model with *self-friends behavioral difference* regressed on mean-centered homophily and participant race, along with their interaction. We found that students of color tended to report behaving more differently from their friends than white students in our sample (*b* = 0.31, *SE* = 0.061, *t*(1168) = 5.048, *p* < 0.001, η^2^ = 0.02, CI = [0.189, 0.43]). Race moderated the relationship between racial homophily and *self-friends behavioral difference* (*b* = 0.199, *SE* = 0.06, *t*(1168) = 3.02, *p* < 0.001, η^2^ = 0.009, CI = [0.081, 0.318]). Not surprisingly, for white students, having more white friends predicts greater perceptions of similarity to one’s set of friends. Surprisingly, for students of color, having more same-race friends predicts *reduced* perceptions of behavioral similarity to one’s set of friends (Fig. 5). Students of color with racially similar networks rate their behavior father from those friends than their white counterparts with racially similar friends. Notably, when controlling for race, homophily did not independently predict *self-friends behavioral difference*. None of these patterns held in an identical model predicting *self-friends values difference*.

People misperceive the average member of their wider community, partly because they are drawing on their closer networks to make such judgments. In exploratory models, we ask if self-friends differences mediate the relationship between social identity and self-average differences. The negative indirect effect of race on *self-average difference* via *self-friends difference* was significant for behaviors and values. The data are compatible with a mediation model in which, for students of color, perceived differences in behavior or values from their friends leads to greater differences from the average peer. This pattern for behavior differences reversed for first-generation and transfer students (See Supplementary Materials, Fig. 6). We did not find *self-friends values difference* playing a mediating role between *self-average values difference* and any other measured identities.

### Behavior and value congruence predicts belonging

**Figure 6:**
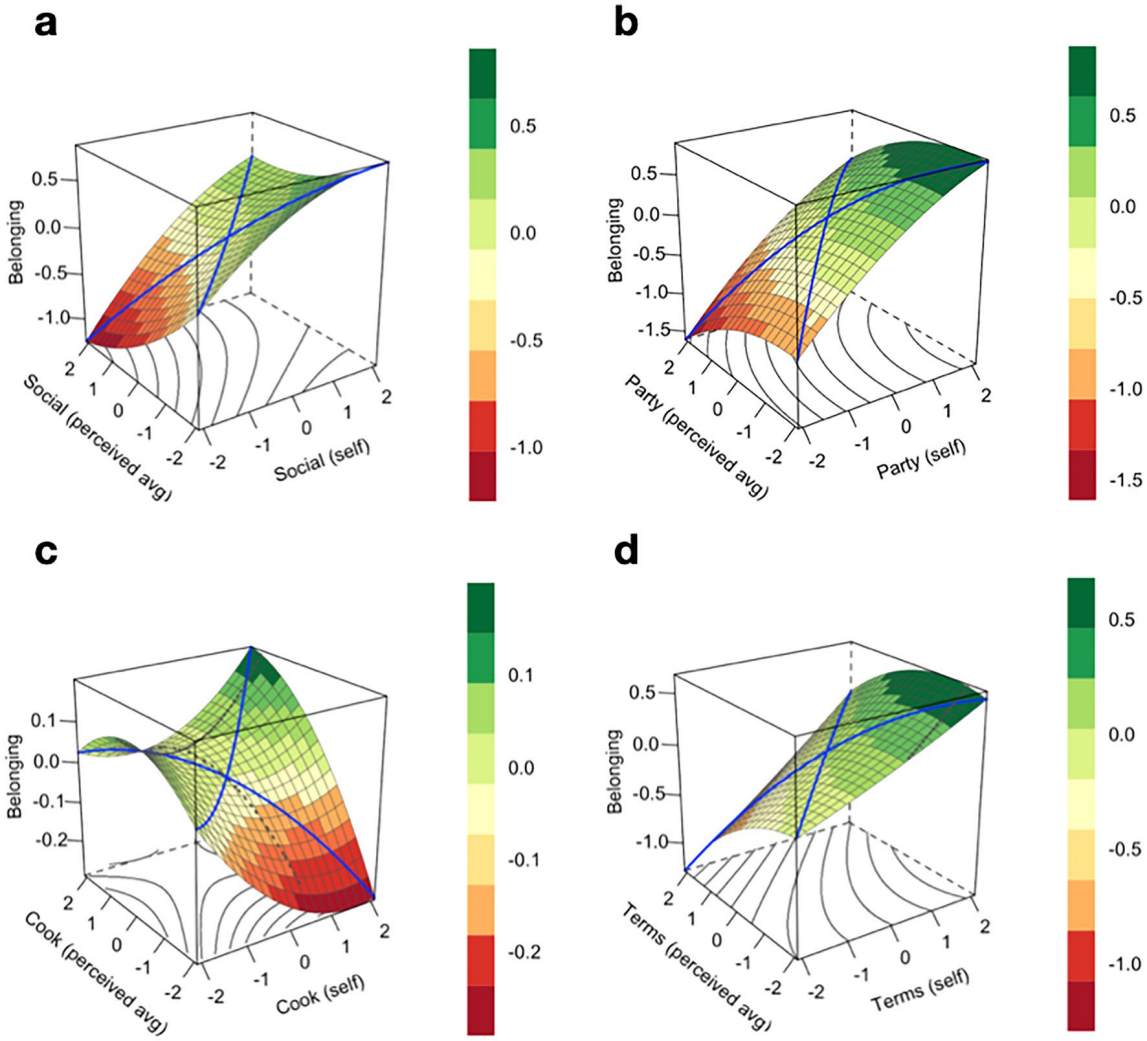
Associations between behavior and belonging are illustrated with four response surface plots based on polynomial regressions predicting belongingness (z-axis) using ratings of behavioral frequencies for oneself (x-axis) and the average student (y-axis). Each regression includes the quadratic terms for each predictor and the two-way interaction of linear terms. Each plot examines one of the four behavioral factors we calculated using an exploratory factory analysis (for details, see Supplementary Materials): (**a**) non-partying social behavior (e.g., attending acapella shows), (**b**), partying social behavior (e.g., going to well-known party locations), (**c**) cooking, driving, and going to the grocery store behavior, and (**d**) language behavior (e.g., students using university-specific terms). For model estimates, including models comparing self-friend and average-friend congruence on these behavioral factors, see Supplementary Materials, Table 8.

**Figure 7:**
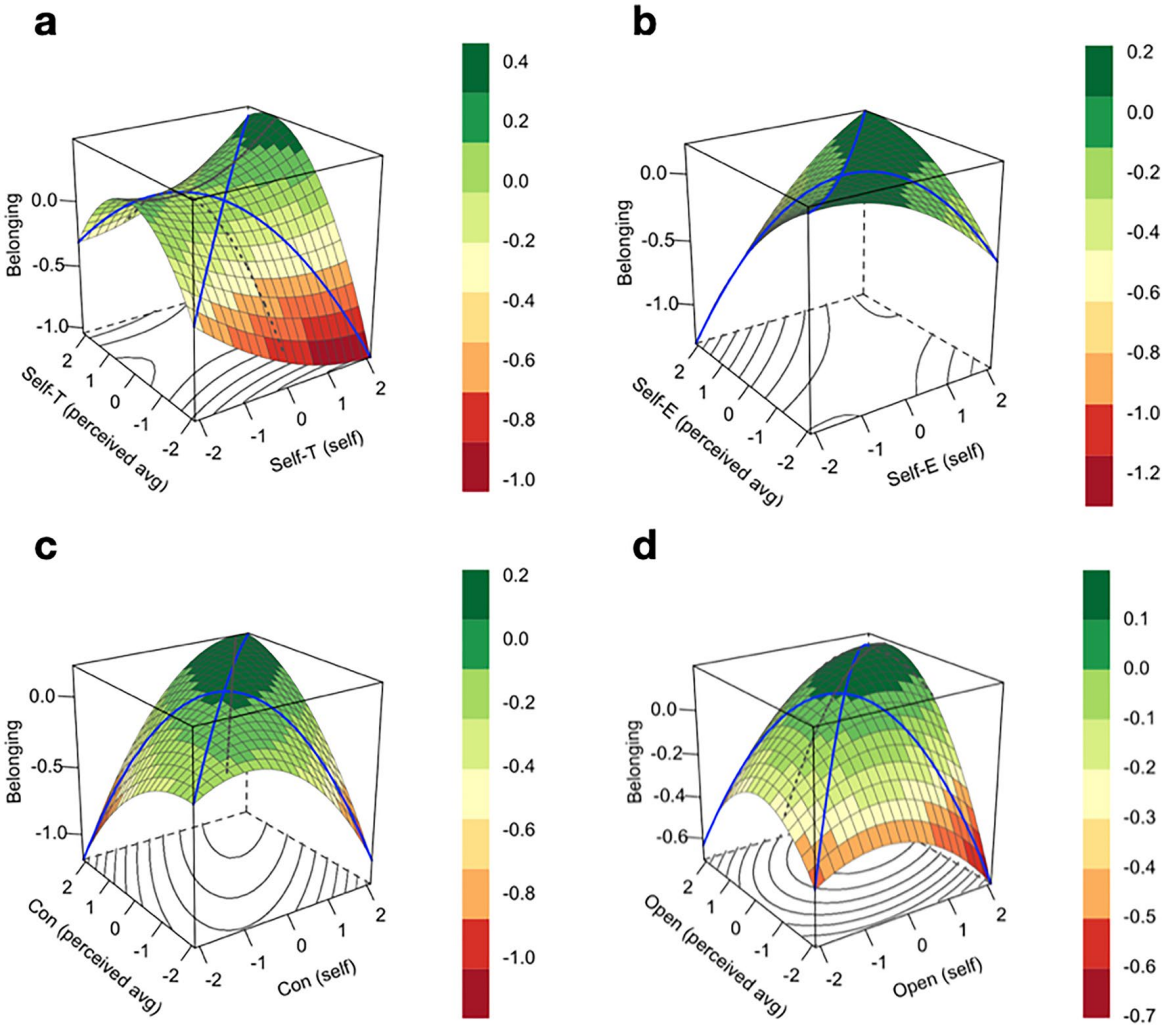
Associations between values and belonging are illustrated with four response surface plots based on polynomial regressions predicting belongingness (z-axis) using value ratings for oneself (x-axis) and the average student (y-axis). Each regression includes the quadratic terms for each predictor and the two-way interaction of linear terms. Each plot examines one of the four Schwartz dimensions: (7a) self-transcendence, (7b) self-enhancement, (7c) conservation, and (7d) openness to change. For model estimates, including models comparing self-friend and average-friend congruence on these value dimensions, see Supplementary Materials, Table 8.

Thus far, our analyses have shown that belonging is related to self-average, self-friends, and average-friends differences, among others. We have consistently found that greater global differences in behavior or values predict lower belonging. These analyses focused on measures of Euclidean difference, which ignore directionality. They cannot tell us if it matters whether a student is (or thinks they are) more or less engaged in behaviors, or hold values more or less strongly, than their peers. We therefore specified exploratory polynomial multiple regression models. Each model predicted belonging from the following standardized predictors: (a) a linear term for self-endorsed values or behaviors (b) a linear term for the perceived average student’s behavior or value endorsement, (c) their quadratic terms, and (d) the two-way linear interaction. We analyzed behavior (Fig. 6) and values (Fig. 7) separately and present just models examining self-average congruence. For model estimates, including models comparing self-friend and average-friend congruence, see Supplementary Materials, Table 8.

To determine whether certain behavior or values categories matter more for belonging, we ran separate models for each of the four behavioral factors that emerged from an exploratory factory analysis (for details, see Supplementary Materials): non-partying social behavior (e.g., attending acapella shows); partying social behavior (e.g., going to well-known party locations); cooking, driving, and going to the grocery store; and language use (e.g., using university-specific terms). We also ran separate models for each of the dimensions underlying the 10 Schwartz values (self-transcendence, self-enhancement, conservation, and openness to change). These models tell us whether belonging is predicted positively or negatively by specific behaviors/beliefs, and whether those associations are moderated by (perceived) peers’ behaviors/beliefs. The quadratic terms test whether more/less of a belief or behavior is always better for belonging, or whether there are diminishing returns.

The regressions reveal an asymmetrical pattern for most of our behavioral factors, apart from cooking-related behaviors (Fig. 6c). A self-average difference where the perception of the average peer’s endorsement of a behavior is high while self-endorsement of that value/behavior is low yields the lowest belonging (Fig. 6a,b,d). In other words, low belonging is most associated with perceiving others to be participating in various social behaviors while you do not. Conversely, the model predicts greater belonging when the perception of the average student’s endorsement is low, while self-endorsement is high (Fig. 6a,b,d). Highly endorsing social behaviors while believing other community members do not generate a greater sense of belonging.

To investigate self-average value congruence, we specified models identical to those examining behavior congruence, using the four conceptual dimensions of Schwartz’s values: self-transcendence, self-enhancement, conservation, and openness to change. Self-transcendence (e.g., universalism and benevolence) positively predicts belonging when someone strongly endorses that value and believes the average peer also endorses it (dark green portion of Fig. 7a). If someone perceives that the average peer does not endorse self-transcendence values, which may be threatening, they report lower belonging, regardless of their own endorsement.

Self-enhancement (e.g., achievement and hedonism) is the conceptual foil to self-transcendence, and we accordingly see a different relationship to belonging (Fig. 7b). Self-average alignment in this value predicts higher belonging regardless of the absolute level. If someone weakly endorses self-enhancement but believes the average peer strongly endorses this value, they report lower belonging—presumably it is threatening to be surrounded by individuals who strongly seek power, especially if their values clash with one's own.

This pattern replicates for valuing conservation (e.g., tradition and conformity; Fig. 7c). Self-average alignment in openness to change (e.g., stimulation and self-direction) optimally predicts belonging when it is moderately or strongly endorsed (Fig. 7d). People report lower belonging when they believe the average peer weakly values openness to change, no matter their own endorsement level. Overall, these analyses demonstrate the differential effects of value congruence on belonging—alignment between self-average endorsement depends on the value itself. Further, we see that many of these relationships are nonlinear, which our preregistered analyses otherwise missed.

## Discussion

The present preregistered work found that the similarity between students' self-reported behavior and values and their perceptions of an “average student” at their university predicted belonging, even when considering their true similarity to the sample and the accuracy of their beliefs about the average. We occasionally found the latter two variables contribute to belongingness, but these effects were smaller and less stable when compared to perceived self-average differences. We further demonstrated that perceived differences in behavior were more influential than those for abstract values: behavioral differences were more consistently related to social identity, belonging, and network structure. Perceptions of behaving differently from peers partially mediated the effect of holding a minoritized identity on belongingness—and these perceptions mattered more than truly behaving differently.

Previous work indicates that discrepancies between an individuals’ values and those of their group causally diminish identification with that group^44^. We add to this understanding by comparing the relative roles of perceived (self-average) differences, actual (self-sample) differences, and accuracy around group norms (average-sample). This work suggests that altering misperceptions of difference may be a better route to improving sense of belonging than the challenging task of modifying actual behavior^48^. Correcting misperceptions may be particularly powerful for improving belongingness among community members with marginalized identities.

We found that perceived *behavioral* differences were more robustly related to belonging, social identity, and network structure than perceived *value* differences. It is possible that the visibility of behavior, compared to values, is driving these results^19^, especially given that values guide behaviors indirectly^52^. In supplementary exploratory analyses, we found that perceived similarity to peers on university-specific behaviors mattered more for belonging than perceived similarity on generic behaviors (See Supplementary Materials). This suggests that when it comes to feeling a sense of belonging to a community, it is most important that you believe you are similar to group members in terms of concrete and identity-congruent behaviors rather than abstract values.

Perceived dissimilarity from the average peer has asymmetric impacts on belonging. Through exploratory polynomial multiple regression models, we demonstrated that students report high belonging when they strongly endorse behaviors while believing other community members do not. The opposite is true, where believing peers highly endorse behaviors while not doing so yields low belonging. We further found differential effects of value congruence on belonging—the consequences of self-average alignment depends on the value itself. These analyses extend previous research on wellbeing and community value similarity^47^ to perceptions of a hypothetical peer.

Perceived similarity to friends predicted belonging independently of perceived similarity to the larger student population, and those two perceptions were only moderately correlated. This work parallels developmental psychologists’ work on similarity between friends underpinning adolescent belonging^53^, but extends it by comparing and controlling for the role of similarity to the average peer. We also found that when students think their friends are unlike the average student, they report reduced belonging, regardless of how similar they think they personally are to the average student. People often resemble their friends^30,32,51^, so having friends deviating from the average might make students feel distinct too.

People with dense and interconnected networks, where their friends all know one another, have greater social support^5^ and stronger peer influence through echo-chamber effects^36,37^. In the present work, students with denser networks reported greater belonging^4^ and also perceived their own behavior to be more similar to their friends’ and peers, compared to students with sparsely-connected networks. However, densely-connected students who believe their friends engage in different daily behaviors than them reported lower belonging than students who believe their friends behave similarly to them.

Since (perceived) behavioral similarity might be driven by identity similarity^54^, and same-race friends typically report more shared daily activities^33^, we asked if our measures of perceived similarity to friends might be predicted by the racial homophily of students’ social networks. We found that when white students have mostly white friends, they perceive themselves to be more behaviorally similar to their friends, compared to white students with more racially diverse friends. This is in line with predictions from the social sampling model, since people make judgments on social information using environmental cues, including homophily^23^. Contrary to our hypotheses, we found that students of color who named more friends with their own racial identity (Asian, Black, multi-racial, or Native American) have lower perceived behavioral similarity with those friends. Some students of color may sacrifice having friends who engage in similar behaviors, choosing instead to prioritize friends who share their racial identity. In this predominantly white institution, students of color have fewer same-race others to select as friends at their university, making it less likely that they will find same-race friends who share all their behavioral tendencies. Future work should test these effects in contexts that differ demographically, such as at Historically Black Colleges and Universities.

This work is not without limitations, many of which will guide future research. This research relies heavily on self-reported behavior; future research might record true behaviors using ecological momentary assessment. Our results are also correlational and cross-sectional. Future work should test if findings hold longitudinally; within-subjects data would more robustly answer these questions, since recent work conceptualizes belonging as an emergent and dynamic feeling^55^.

Since the true population base rates are unknown for the behaviors and values of interest, we used the sample means as proxies for the population mean. However, our sample was less racially diverse, younger (sample was eligible for study if enrolled in their first psychology course) and had fewer male-identifying students compared to the greater university population (See Supplementary Materials; Sample representativeness). Future research should representatively sample across social identity and school year to obtain a sample reflective of the entire university.

Future interventions should target misunderstandings about typical peer behavior and individuals’ perceived behavioral dissimilarity to peers. Misperceptions surrounding others’ behavior or attitudes is widespread across domains^56^. Interventions can modify these descriptive norm misperceptions^40,57^. Prior work has reduced identity-based gaps in belonging by intervening on behavior^46^. But manipulating behavior long-term can be difficult^48^. Rather than encouraging behaviors to help people “fit in,” interventions can help individuals realize the commonalities they share with the rest of the community.

## Method and materials

All study materials, data, and analysis scripts are openly available on OSF (https://osf.io/wv5r6/?view_only=b812a856a3944cc190e556ac0678501d).

### Participants and procedure

Participants (N = 1181) were recruited through the psychology participant pool at a large mid-Atlantic university in the United States for a 30-min online Qualtrics study in Fall 2022. All measures assessed are reported; those not analyzed in the main text are in Supplementary Materials. Participants gave informed consent prior to the study and were compensated with course credit after the debriefing. We aimed to recruit as many students as possible in a semester, thus the sample size was not determined based on a power analysis. The University of Virginia Institutional Review Board approved this research. All data collection and experimental processes ethically followed this approved protocol and regulations from the Declaration of Helsinki.

We included participants’ data if they completed the survey and had less than 10% of data missing, excluding 68 participants from the original sample of 1249. Participants were over 18, with an average age of 18.7. See Table 1 for sample characteristics relevant to college belonging.

**Table 1:** Sample Demographic Characteristics.

Identity	% of N
Racial Identity	
White	60.03
Asian American	22.02
Black / African American	6.35
Other	1.86
Multiracial	.89
Hawaiian Native / Pacific Islander	.1
Gender Identity	
Cis Female	66.98
Cis Male	29.63
Non-binary	.76
Other	.89
Gender fluid	.51
Trans male	.08
Trans female	.08
Out of state	36.24
International students	5
First-generation students	14.31
Transfer students	7.87

### Measures

#### Demographic Information

Participants reported the number of semesters they have spent in [city], their living accommodations, and if they were in-state, first-generation, international, or transfer students.

Participants reported their racial identity. Because we had so few participants in several categories and given that the majority or ‘average student’ is white, we coded race as 0.5 = students of color (39.96%), and − 0.5 = white students, to use data from all students.

Participants reported their household income. To combat the heavy skew within our pilot sample (See Supplementary Materials, Fig. 13), we measured income with open-ended text entry; unfortunately, 40.2% of our sample did not answer the question, and many others provided nonsensical responses (e.g., “0”, “12”). For this reason, we exclude this variable when analyzing social identity.

#### Social network structure

Students reported their social network with a name generator task: “Consider the people with whom you like to spend your free time. Since you arrived at [university], who are the classmates you have been with most often for informal social activities, such as going out to lunch, dinner, drinks, films, visiting one another’s homes, exercising together, and so on?”^58^. Students could list up to 100 social ties but saw five fields at a time. After listing those friends’ names (M = 5.03, SD = 1.72, min. = 0, max. = 27), they report each friend’s racial/ethnic identity, gender, and level of closeness (0–100, 100 = very close). Students then reported which of their friends knew one another.

#### Social network metrics

We used students’ reports of which friends were connected to which to calculate their ego-centric *network density* (M = 0.63, SD = 0.3, min. = 0, max. = 1), or the number of ties divided by the number of possible ties. This captures the degree of interconnectedness among one’s ties. We also calculated their networks’ *racial homophily* (M = 0.6, SD = 0.35, min. = 0, max. = 1) as the proportion of friends with the same race as the participant.

#### Belongingness

The College Belongingness Questionnaire assessed belonging (Asher & Weeks, 2013). Participants were asked “How well do you think you belong at UVA?” and rated the extent to which they agree (1 = strongly disagree; 5 = strongly agree) with statements like “I feel connected to this school.” The items were appropriately reverse-coded and averaged to be used in analyses (*M* = 3.91, *SD* = 0.84).

#### Behavior

Participants reported how frequently they, their friends, and a hypothetical “average student” engaged in local behaviors. These items were generated by 14 undergraduate research assistants who were asked “What are the norms, trends, activities, preferences, and behaviors that are specific to [university]?” The resulting 18 items described the frequency of engaging with a particular location or behavior (1 = never; 6 = daily). Example local behaviors include going to the local bagel shop and having a picnic at popular campus locations. We also asked participants for the frequency with which they, their friends, and a hypothetical average student engage with 16 behaviors unattached to a particular place (1 = never; 6 = daily). Example general behaviors include cooking food at home, doing homework, and exercising (See Supplementary Materials, Tables 1 and 2 for full behavior lists).

#### Values

Participants reported values for themselves, their friends, and those of a hypothetical average student using the Short Schwartz’s Value Survey^59^. These 10 values were rated on a Likert scale (0 = opposed to my principles; 8 = of supreme importance), to reflect how strongly participants believed themselves or others to hold that value. The assessed values included power, achievement, hedonism, stimulation, universalism, benevolence, tradition, conformity, security, and freedom (for descriptive statistics, see OSF).

#### Difference Scores

We calculated the Euclidean distances between participants’ self- and other-reported behavior and values. Item by item, we calculated the difference between (a) each participant’s self-score and their score for an average student (self-average difference), (b) each participant’s self-score and the sample’s mean score (self-sample difference), () (c) their score for an average student and the sample’s mean score (average-sample difference), (d) each participant’s self-score and their score for their close friendship network (self-friends difference), and (e) their score for an average student and their score for their friendship network (average-friends difference). Separately for behavior and values, we then squared the item-wise differences, summed them, then took the square-root. Euclidean distance has a relatively straightforward interpretation and is appropriate for data with scaled dimensions^60^. The result was 10 difference scores (Fig. 1), with equations for behaviors below:

- *Self-average behavioral difference* = $\sqrt{{\sum }_{n=1}^{34}({s}_{{behavior}_{i}}-{p}_{{behavior}_{i}})}$^*2*^
- *Self-sample behavioral difference* = $\sqrt{{\sum }_{i=1}^{34}({s}_{{behavior}_{i}}-{a}_{{behavior}_{i}})}$^*2*^
- *Average-sample behavioral difference* = $\sqrt{{\sum }_{i=1}^{34}({p}_{{behavior}_{i}}-{a}_{{behavior}_{i}})}$^*2*^
- *Self-friends behavioral difference* = $\sqrt{{\sum }_{n=1}^{34}({s}_{{behavior}_{i}}-{f}_{{behavior}_{i}})}$^*2*^
- *Average-friends behavioral difference* = $\sqrt{{\sum }_{n=1}^{34}({p}_{{behavior}_{i}}-{f}_{{behavior}_{i}})}$

Where ${s}_{{behavior}_{i}}$ is a participant’s report of how frequently they engage in normative behavior *i,* ${a}_{{behavior}_{i}}$ is the sample mean of behavior *i* engagement, ${p}_{{behavior}_{i}}$ is a participant’s estimate of how frequently they think the average peer engages with behavior *i*, and ${f}_{{behavior}_{i}}$ is a participant’s estimate of how frequently they think their friends engage in behavior *i.*

**Figure 8:**
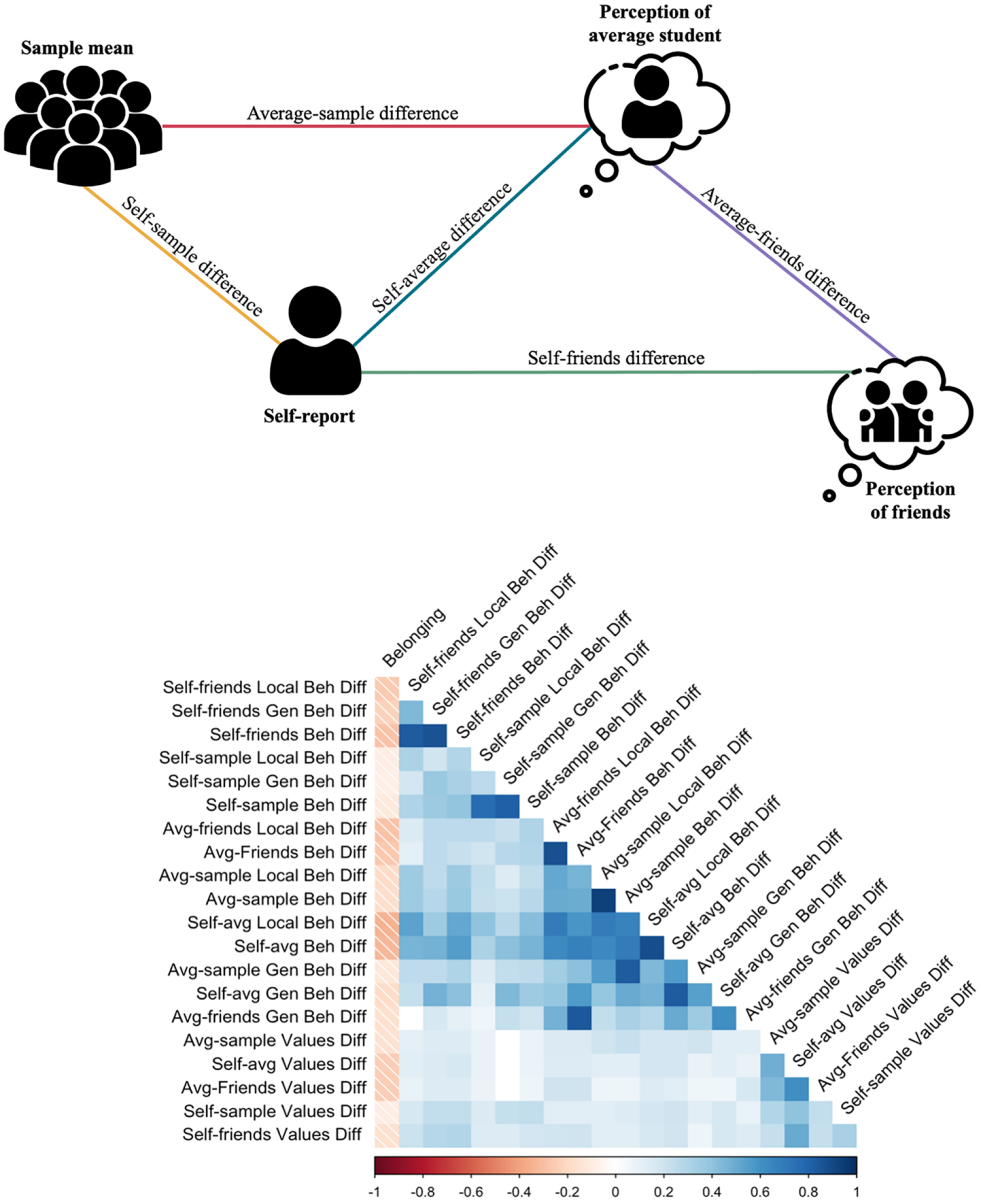
Difference scores calculated separately for behaviors (local and general behaviors together) and values, presented along with their bivariate correlations with belonging. For descriptive statistics, histograms, and correlations of all unstandardized difference scores, see Supplementary Materials, Figs. 1,  2, and Table 4.

The *behavioral difference* scores are the Euclidean distances for all 34 behaviors, general and local behaviors combined. We calculated our remaining difference scores for analyses by adapting the above equations for the 10 Schwartz’s values for a total of 10 differences. These scores are meant to capture differences in either behaviors or values across multiple dimensions, rather than differences on a single item^26^. See Fig. 8 for a conceptual diagram and correlation matrix relating each of these difference scores.

We conducted exploratory analyses (not preregistered) with behavioral difference scores for local and general behaviors separately, because they were not all highly correlated (see Supplementary Materials).

All difference scores were centered and standardized for analyses using the scale() function in R. See Supplementary Materials (Figs. 1, 2, Table 4) for descriptive statistics and histograms of the unstandardized difference scores.

### Analytic strategy

Unless otherwise noted (marked as “exploratory”), all analyses were preregistered on OSF (https://osf.io/wv5r6/?view_only=b812a856a3944cc190e556ac0678501d). We conducted a linear regression in R predicting belonging with all difference scores as predictors. To examine social identity variability in belongingness and difference scores, we then ran exploratory linear regressions using categorical variables for racial, international, in-state, first-generation, and transfer statuses as predictors, controlling for semesters spent on campus.

We used the social identity factors that significantly predicted belonging as predictors in preregistered mediation models, with behavioral and value differences as mediators and belonging as the outcome. For our mediation analyses, we utilized the sem() function from the lavaan R package^61^. We then conducted preregistered linear regression models examining the relationships between density, differences from friends, and belonging, as well as racial homophily, racial identity, and differences from friends.

For preregistered analyses with additional variables—feeling different from the average, total closeness to one’s social network, and mediation models involving self-friends differences—and exploratory analyses isolating local and general behaviors, see Supplementary Materials. For complete data and exploratory analyses with loneliness as the key outcome, see OSF.

All preregistered analyses utilize Euclidean difference scores. However, these scores are agnostic to directionality. We might question whether it matters for belongingness if someone holds values or endorses behavior more or less intensely than the average student or their network. To test this question, we specified several polynomial multiple regression models examining the relationship between behavior or value ratings for oneself and the perception of either the ‘average student’ or their friends. Previous work investigating environment fit in values and wellbeing has employed this approach^47^. We estimated 2 (values and behavior) × 4 (value dimension and behavioral factor) × 3 (self-average, self-friend, and average-friend) models. We cannot compute parallel analyses examining self-sample congruence, since all participants have identical values and models do not converge. We focus on reporting models examining self-average congruence, which are visualized using response surface plots using the plotRSA() function in the RSA R package^62^. For model estimates, including models comparing self-friend and average-friend congruence, see Supplementary Materials, Table 8.

### Supplementary Information

Supplementary Information.

## Data Availability

Complete data processing code, complete analysis code, study materials, pre-registrations, and de-identified data are available on OSF (https://osf.io/wv5r6/?view_only=b812a856a3944cc190e556ac0678501d).
