# Supplementary material for: Thinking you're different matters more for belonging than being different
Source: Sci Rep. 2024 Mar 30;14:7574. doi: 10.1038/s41598-024-58252-y (PMC10981754; doi:10.1038/s41598-024-58252-y)
Supplement: Supplementary file 1 — Supplementary Information. [file 41598_2024_58252_MOESM1_ESM.pdf]

Supplementary Materials

Thinking you're different matters more for belonging than being different

Sareena Chadha[1*], Tiffany Ha[1], & Dr. Adrienne Wood[1]

Supplementary Materials for Main Study
University-specific Behavior List

Go to Bodo's Bagels	Streak the Lawn
Go to Culbreth Garage for Sunset	Go out during the week
Go to the Corner at night	Feel lonely*
Go to Beta Bridge	Use the term 'Grounds'
Go to Mad Bowl	Use terms like 1st year, 2nd year, etc.
Attend football games	Pull all-nighters in the library
Go to acapella shows	Hear other talk about NoVa
Get food from amphitheater food trucks	Feel homesick*
Hike	Wear khakis or tennis skirts
Feel like they're not doing enough*	Get lost*
Have a picnic on the Lawn/Grounds	Go to a dining hall

Table 1. *Full list of university-specific behaviors which described either a) event or location attendance (1 = extremely unlikely; 6 = extremely likely) or b) frequency of engaging with a particular behavior or feeling (1 = never; 6 = daily). * = only in pilot study*

General behavior list

Exercise	Drink
Cook food at home	Play video games
Eat food out at restaurant or fast-food place	Play organized sports (IM to D1)
Go to the grocery store	Play recreational sports (frisbee or spikeball with friends)
Watch TV	Be with family

Read a book for pleasure	Drive
Go for a walk or run	Study / do homework
Clean your living space	Go out with friends

Table 2. *Full list of general behaviors which described either a) event or location attendance (1 = extremely unlikely; 6 = extremely likely) or b) frequency of engaging with a particular behavior (1 = never; 6 = daily).*

Sample representativeness

The sample is fairly representative of the 2020 undergraduate population at the university, except for racial and gender identities. The sample is racially imbalanced when compared to the overall undergraduate student population.

**Race**	**Population** (*Undergraduate Students…*, 2020).	**Sample**
White non-Hispanic	55.67%	60.03%
Asian	16.16%	22.02%
African-American / Black	6.74%	6.35%
Hispanic / Latinx	6.73%	
Multi-racial	5.16%	0.89%
Unknown	5.13%	1.86%
Non-resident alien	4.24%	
Native American / Alaskan	.08%	
Native Hawaiian / Pacific Islander	.08%	.1%

Table 3. *Racial demographics of sample and population.*

Table 3 reflects a slight overrepresentation of two main racial groups in the sample (White and Asian), meaning there was less racial diversity in the sample than in the population overall. The undergraduate student population is 56% female and 44% male (*College Navigator…*, 2022),

Measures not analyzed in the main text

Feeling Different. To measure the subjective feeling of being different, we simply asked participants "How different do you feel from the average [university] student?", with responses on a sliding scale (0-100, 100 = very different from the average; *M* = 47.31, *SD* = 22.29).

Total Closeness. An additional social network metric we calculated was *total closeness* (M = 374.03, SD = 147.64, min. = 0, max. = 1280), as the sum of all closeness scores for each friend nominated.

Loneliness. We assessed loneliness with the UCLA Loneliness 8-item scale (Russell, 1996). Students were asked how they identify with statements like “I feel left out”. All items were assessed on a 4-point Likert scale (1 = never; 4 = often). After reverse-coding appropriately, we sum the items to be used in analyses (M = 16.67, SD = 4.87, min. = 8, max. = 31). Analyses treating loneliness, rather than belonging, as the dependent variable can be found in the OSF output.

Interpersonal Similarity. We adapted the Interpersonal Similarity Scale (Schug et al., 2009) for students to report on how similar they and their friendship network are (rather than just a close tie). We asked “How similar are you and your close friends (named earlier in the survey) on the following items?” with items like personality, behavior, and values (1 = not similar at all; 7 = very similar). Analyses examining the behavior and values single-item questions predicted by all difference scores can be found in Supplementary Materials.

Difference scores for local and general behaviors in isolation. Figure 1 shows the histograms for the differences scores in the current study before they were centered and standardized for analyses.

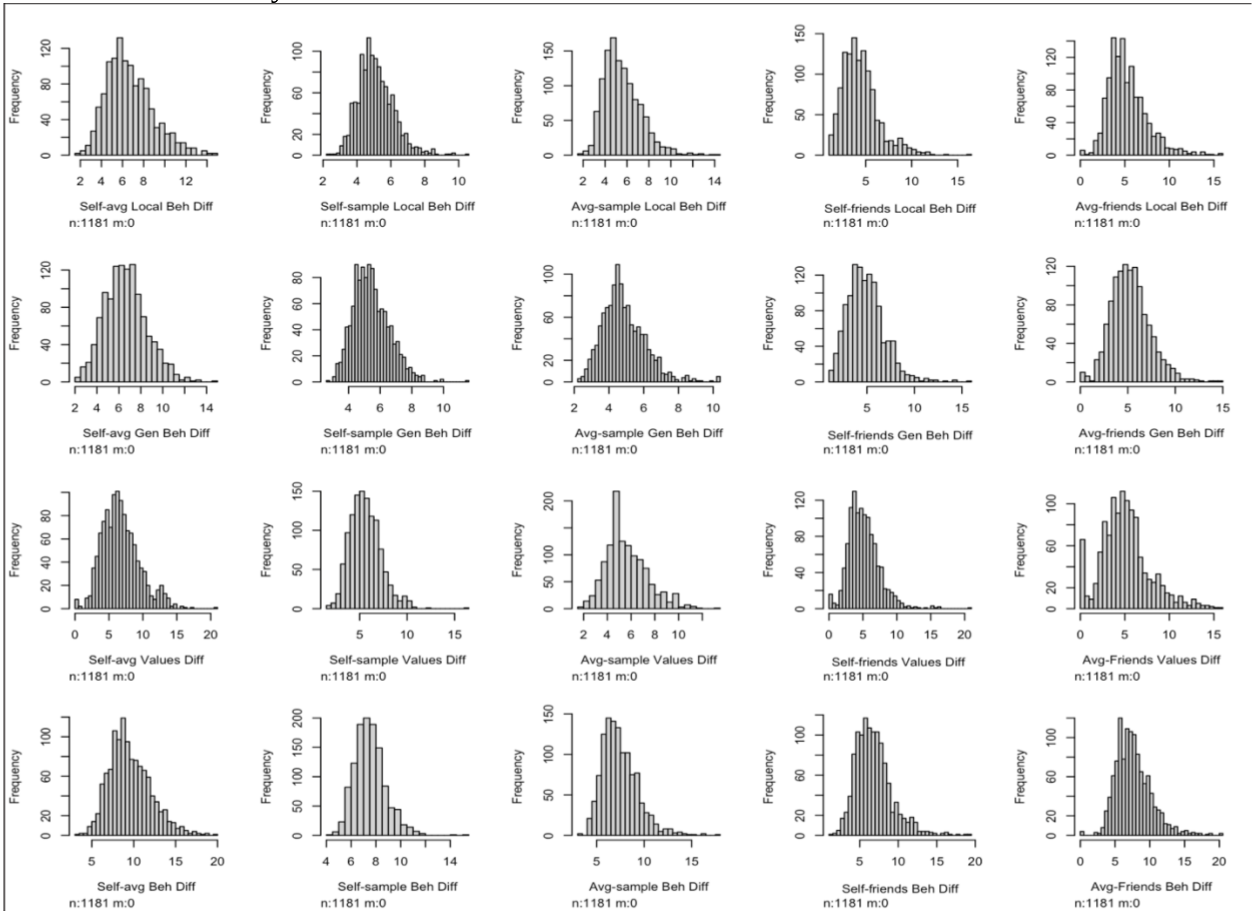

Figure 1. *Histograms of the raw difference scores.*

Descriptive statistics for difference scores. Table 4 (below) summarizes the descriptive statistics for the unstandardized Euclidean difference scores. Figure 2 expands on Figure 8 in the main text.

Difference Score	Mean	SD	Min.	Max.
Average-friends Behavioral Difference	7.676	2.566	0.000	20.421
Average-friends General Behavioral Difference	5.323	2.029	0.000	14.799
Average-friends Local Behavioral Difference	5.320	2.182	0.000	15.716
Average-friends Values Difference	5.110	2.734	0.000	15.556
Average-sample Behavioral Difference	7.439	1.868	3.134	17.620
Average-sample General Behavioral Difference	4.855	1.226	2.244	10.341
Average-sample Local Behavioral Difference	5.559	1.689	1.957	14.267
Average-sample Values Difference	5.606	1.723	1.792	13.407
Self-average Behavioral Difference	9.580	2.483	3.162	19.698
Self-average General Behavioral Difference	6.659	1.889	2.236	14.697
Self-average Local Behavioral Difference	6.725	2.193	1.732	14.731
Self-average Values Difference	6.782	2.744	0.000	20.905
Self-friends Behavioral Difference	6.826	2.372	1.414	19.209
Self-friends General Behavioral Difference	4.932	1.917	1.000	16.000
Self-friends Local Behavioral Difference	4.507	1.977	1.000	16.062
Self-friends Values Difference	5.173	2.269	0.000	20.616
Self-sample Behavioral Difference	7.530	1.244	4.029	15.031
Self-sample General Behavioral Difference	5.440	1.133	2.783	11.426
Self-sample Local Behavioral Difference	5.124	1.056	2.287	10.556
Self-sample Values Difference	5.617	1.617	1.866	16.483

Table 4. *Descriptive statistics for all difference scores, used in both preregistered and exploratory analyses.*

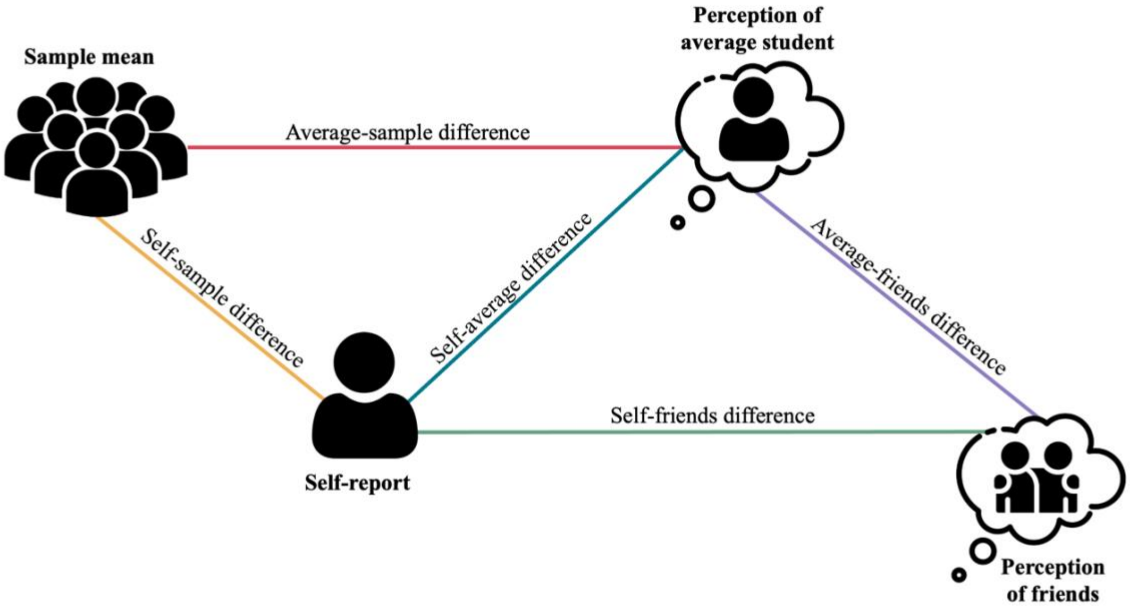

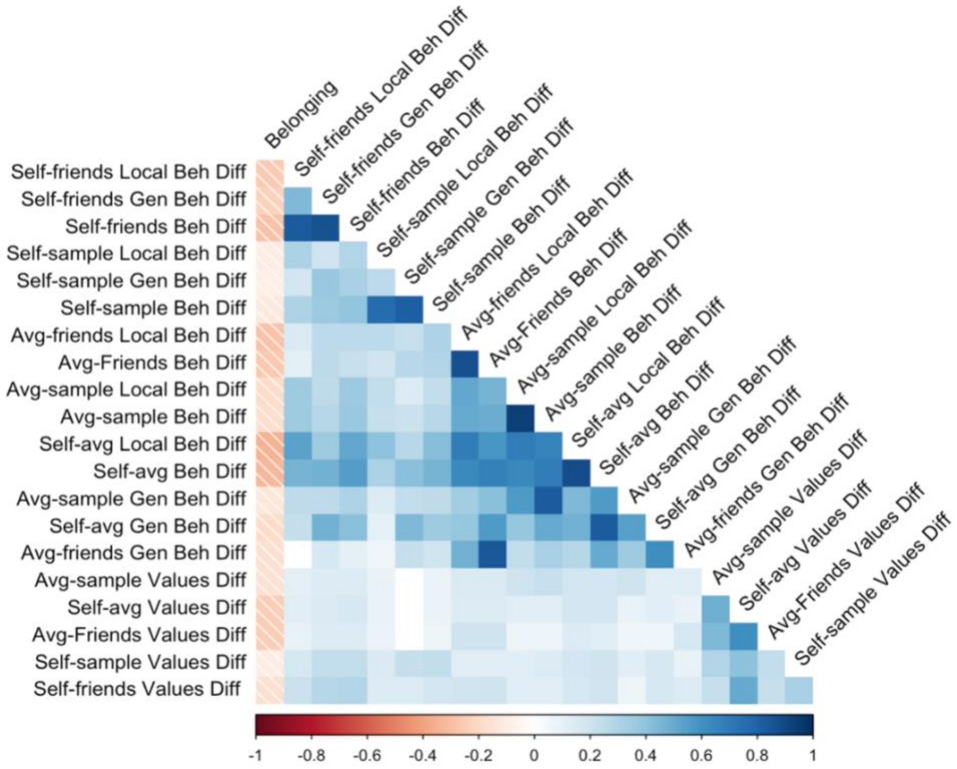

Figure 2. *Bivariate correlations between belonging and all difference scores, used in both preregistered and exploratory analyses. We present behavioral difference scores for the subsets of local and general behaviors together and separately. Figure 8 in the main text only examines behavioral differences using all self-reported behaviors.*

Behavior and value differences predict feeling different from others

In addition to our key dependent variable, belonging, we also ask which difference scores best predict participants' feeling different from their peers in a global sense. We specified a model with the ten difference scores predicting feeling different from the average student (Figure

3). For this first model, we adhered to the preregistered plan to use our global behavioral difference scores, rather than the separate difference scores for general and local behaviors. We followed this up with post hoc analyses in which we separate general and local behaviors.

We found that *self-average behavioral difference* positively predicted feeling different from the average (b = 2.957, SE = 1.192, t(1170) = 2.48, p =.013), such that people who rated the average student's behavior like their own felt more like the average. We additionally found that *self-average values difference* positively predicted feeling different from the average (b = 3.041, SE = .955, t(1170) = 3.184, p = .001). Those who rated their values in line with their perception of the average student's values felt more similar to the average. We also found that *self-sample behavioral difference* positively predicts feeling different from the average (b = 2.693, SE = .722, t(1170) = 3.728, p <.001); students who rated their behavior far from the sample mean felt more different from the average. Neither *self-sample values difference, average-sample differences*, nor the *self-friends difference* measures were significant predictors of feeling different.

We then ask which of these differences predict feeling different from friends' behaviors and values. In two models, we use ten difference scores to predict individual items for "How similar are you and your friends in terms of behaviors?" and "How similar are you and your friends in terms of values?". We found that *self-friends differences* appropriately predicted each of these items, when controlling for all other differences (For these model outputs, see the OSF page); this model serves as a validity check for difference scores involving friends.

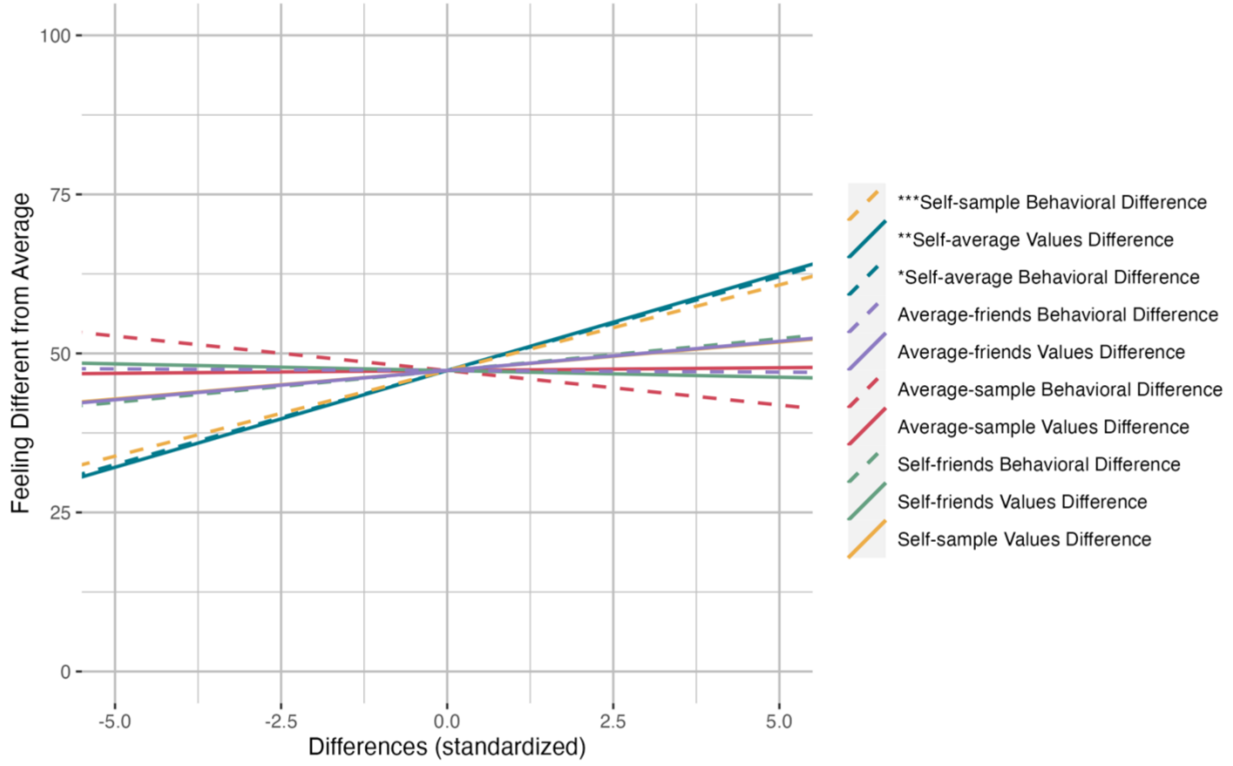

Figure 3. *Model predictions from a regression predicting participants' feelings that they are different from other students using all behavior and values differences scores (standardized). Dashed lines represent behavioral differences, while solid lines represent differences in values. (***p<.001, **p<.01, *p<.05)*

To investigate which types of behavior influence feeling difference from the average student, we then run an exploratory model with self-average, self-sample, average-sample, self-friends, and average-friends differences for a) local behavior, b) general behavior, and c) values as predictors. Of all 12 difference scores, only three were significant predictors. *Self-average local behavioral difference* (b = 3.357, SE = 1.27, t(1165) = 3.926, p = .008) and *self-average values difference* (b = 3.014, SE = .955, t(1165) = 3.155, p =.002) positively predicted feeling different from average. Students who rated their local behaviors and values far from their perception of an average student felt more different from that peer. *Self-sample general behavioral difference* (b = 2.741, SE = .736, t(1165) = 3.725, p <.001) positively predicted feeling different from the average student when controlling for all other measures of difference. Those who reported general behaviors, or daily habits, that were farther from the sample mean also felt more different from the average student. *Self-sample general behavioral difference, self-sample local behavioral difference, self-sample values difference*, all *average-sample* and *self-friends differences* were insignificant in this model. We find that *self-average general behavioral difference* does not predict feeling different from the average student, but *self-average local behavioral difference* does. This underscores the importance of perceiving oneself as acting similarly to one's community *specifically* in behavioral domains that are central to the community's identity.

Exploratory follow-up analyses of behavioral subtypes

To explore if the local and general subsets of behavioral differences distinctly impact belonging, we specify a non-preregistered model with belonging regressed on self-average, self-sample, and average-sample differences from the average student and self-friends and average-friends differences for a) local behavior, b) general behavior, and c) values. *Self-average local subset behavioral difference* (b = -.25, SE = .046, t(1150) = -5.458, p < .001, η^2 = .025, CI = [-.34, -.16]) and (as before) *self-sample values difference* (b = -.11, SE = .034, t(1150) = -3.181, p = .002, η^2 = .009, CI = [-.177, -.042]) negatively predicted belongingness. Students reported lower levels of belonging if they rated their local behaviors or values differently than those they perceive of the average student. *Self-sample local subset behavioral difference* (b = .056, SE = .025, t(1150) = 2.239, p = .025, η^2 = .004, CI = [.007, .105]) and *self-sample values difference* (b = .052, SE = .025, t(1150) = 2.053, p = .04, η^2 = .004, CI = [.002, .104]) positively predicted belongingness, reflecting the same suppressor effect as in the prior model. Students who rated their local behavior or values far from the sample mean behavior or values reported greater belongingness, when controlling for perceived differences (bivariate relationship is negative). *Average-sample local subset behavioral difference* positively predicted belonging (b = .088, SE = .034, t(1150) = 2.578, p = .01, η^2 = .006, CI = [.021, .154]), such that students who rate their perception of the average student's behavior far from the true sample mean report a greater sense of belonging (another suppressor effect). Belonging was also predicted by *self-friends local subset behavioral difference* (b = -.083, SE = .03, t(1150) = -2.741, p = .006, η^2 = .006, CI = [-.143, -.024]) and *self-friends general subset behavioral difference* (b = -.083, SE = .029, t(1150) = -2.862, p = .004, η^2 = .007, CI = [-.139, -.026]). In other words, students who rated their behaviors, general or local, differently from their friends' reported lower college belonging. Lastly, *average-friends values difference* negatively predicted belonging (b = -.092, SE = .03, t(1150) = -3.067, p = .002, η^2 = .008, CI = [-.151, -.033]); those who rated their friends' and the average students' values far apart reported lower belonging. All other tested predictors, most notably *self-average general subset behavioral difference,* were insignificant.

This model suggests that (perceived) behaviors particularly relevant to one's identification with a community are more central to sense of belonging than matching, or believing oneself to be matching, the behavior of community members more broadly. *Self-average local subset behavioral difference* has a larger effect than *self-average general subset behavioral difference* on belongingness.

Social identity variability in difference scores

Variable	Race	Semesters	In-state	First-generation	Transfer	International
Avg-friends Beh Diff	0.395***	-0.101***	0.043	0.388***	0.16	0.32*
Avg-friends General Diff	0.279***	-0.204***	-0.003	0.247**	-0.204	0.331*
Avg-friends Local Beh Diff	0.392***	0.028	0.077	0.406***	0.448***	0.216
Avg-friends Values Diff	-0.002	0.13***	-0.143*	0.199*	0.148	-0.008
Avg-sample Beh Diff	0.467***	-0.032	0.061	0.471***	0.261*	0.442***
Avg-sample General Beh Diff	0.359***	0.001	-0.007	0.274***	0.091	0.403**
Avg-sample Local Beh Diff	0.45***	-0.046	0.091	0.498***	0.323**	0.398**
Avg-sample Values Diff	0.101	0.081**	-0.021	0.2*	0.21	-0.209
Belongingness	0.303***	0.046	0.07	0.108	0.459***	0.385**
Self-avg Beh Diff	0.34***	-0.031	-0.003	0.094	0.095	0.238
Self-avg General Beh Diff	0.131*	0.113***	0.129*	0.069	0.67***	0.393**
Self-avg Local Beh Diff	0.152*	0.057	-0.075	0.113	0.345**	0.049
Self-avg Values Diff	-0.32***	-0.032	0.142**	-0.291***	-0.371***	-0.025
Self-friends Beh Diff	0.488***	-0.099***	-0.002	0.569***	0.232*	0.379**
Self-friends General Beh Diff	0.411***	-0.227***	-0.005	0.382***	-0.305**	0.302*
Self-friends Local Beh Diff	0.431***	0.036	0.011	0.586***	0.616***	0.339**
Self-friends Values Diff	-0.034	0.095***	-0.22***	0.142	0.221*	-0.041
Self-sample Beh Diff	0.272***	0.015	-0.016	0.373***	0.526***	0.223
Self-sample General Beh Diff	0.305***	-0.01	-0.058	0.265**	0.32**	0.171
Self-sample Local Beh Diff	0.149*	0.039	0.009	0.388***	0.542***	0.27*
Self-sample Values Diff	0.12	-0.014	-0.054	-0.003	0.179	-0.13

Table 5. *Beta coefficients for each social identity facet on belongingness and difference scores. Significant relationships are highlighted in yellow (***p<.001, **p<.01, *p<.05).*

Identifying social identity predictors of belonging to be used in mediation models

We ran a linear regression model with social identity (race and in-state, first generation, international, and transfer student status) predicting belongingness, controlling for semesters spent on campus (Table 5). Race negatively predicted belonging (b = -.32, SE = .051, t(1157) = -6.276, p <.001, η^2 = .033, CI = [-.421, -.22]), such that students of color reported lower levels of belongingness than white students. First-generation (b = -.291, SE = .07, t(1157) = -4.161, p <.001, η^2 = .015, CI = [-.428, -.154]) and transfer student status (b = -.371, SE = .09, t(1157) = -4.11, p <.001, η^2 = .014, CI = [-.549, -.194]) negatively predicted belonging. Students who transferred universities or identified as first-generation students reported lower belonging. In-state status positively predicted belongingness (b = .142, SE = .053, t(1157) = 2.683, p = .007, η^2

= .006, CI = [.038, .245]), meaning students who were out-of-state reported greater belonging compared to state residents. International student status and total semesters spent on campus were unrelated to belonging in this model.

In separate models, we found identity effects on many difference scores (Main Text Figure 3; see *Supplementary Materials Table 5* for model estimates). Generally, students of color, first-generation students, and transfer students have greater differences, across both behaviors and values (Table 5).

Behavioral differences from friends partially mediate social identity effects on belongingness

In addition to differences from the "average" peer, we preregistered analyses asking if self-friends differences mediated the relationship between social identity and belongingness (recall that there are no difference scores involving friends' sample, because we do not know the behaviors and values of each student's friends). We specified models for race, transfer, in-state, and first-generation student status (Figure 4) since these identity facets are related to belonging (Table 5). The negative indirect effect of race on belongingness via *self-friends behavioral difference* was significant (b = -.069, SE = .049, p = <.001, δ = -.069, CI = [-.098, -.04]; a*b path in Figure 4) This finding extends our other mediation results, illustrating differences in behavior, from both the "average student" and from one's friends, reduce belonging in students who hold marginalized racial identities. This pattern replicated for first-generation and reversed for transfer students (Figure 4). We did not find *self-friends values differences* playing a mediating role between belonging and any of our measured identities.

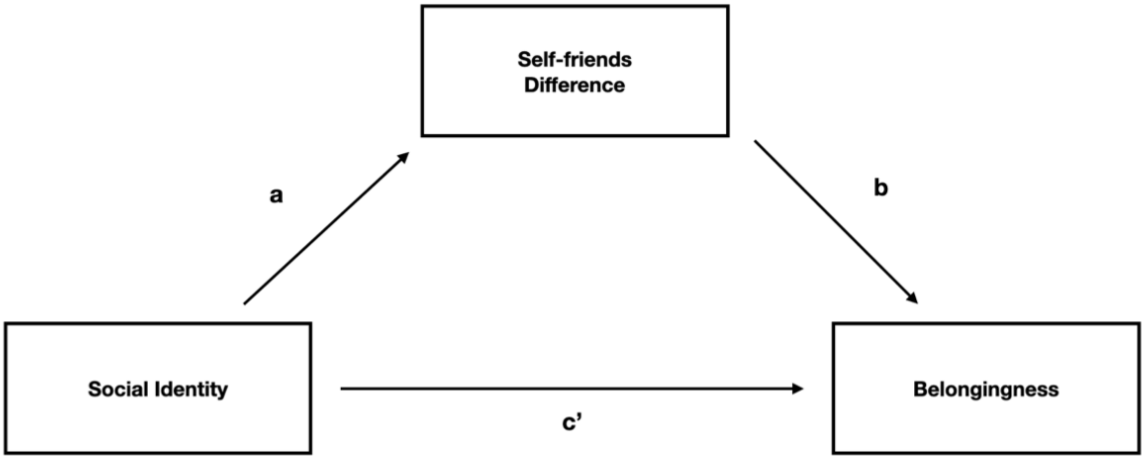

Identity	Type	a	b	cp	ab	total
Race	Behaviors	0.32***	-0.216***	-0.311***	-0.069***	-0.38***
	Values	0.118*	-0.136***	-0.364***	-0.016	-0.38***
Transfer	Behaviors	0.556***	1.079***	1.26*	0.6***	1.861***
	Values	0.198	0.493***	1.763***	0.098	1.861***
First-Gen	Behaviors	0.467***	-0.222***	-0.312***	-0.104***	-0.415***
	Values	0.036	0.511***	1.787***	0.018	1.805***
In-state	Behaviors	-0.085	1.109***	-1.129***	-0.094	-1.223***
	Values	-0.105	0.49***	-1.172***	-0.051	-1.223***

Figure 4. *Structure for each of the mediation models with perceived difference from friends as*

*the mediator, with outputs in table. Highlighted are the significant indirect effects of self-friends behavioral differences that partially mediated the relationship between social identity and belongingness (***p<.001, **p<.01, *p<.05).*

Behavioral differences from friends partially mediate network density effects on belongingness

We specified exploratory mediation models with perceived differences from friends mediating the relationship between network density and belonging, to extend our moderation analyses (See Main Text, Figure 4). We found a significant indirect effect for behavioral self-friend difference, such that those with tight-knit networks rated their behavior close to their friends', and in turn reported lower belonging (b = .028, SE = .006, p = <.001, δ = .028, CI = [.015, .04]; a*b path in Figure 5). The model also yields a positive direct effect of density on belonging, where students with denser friendship circles report greater belonging (Figure 5).

This finding extends our main text results, illustrating the complex relationship between network density, behavioral similarity to one's network, and belonging. We did not find *self-friends values differences* playing a mediating role between belonging and density, mirroring our moderation analyses.

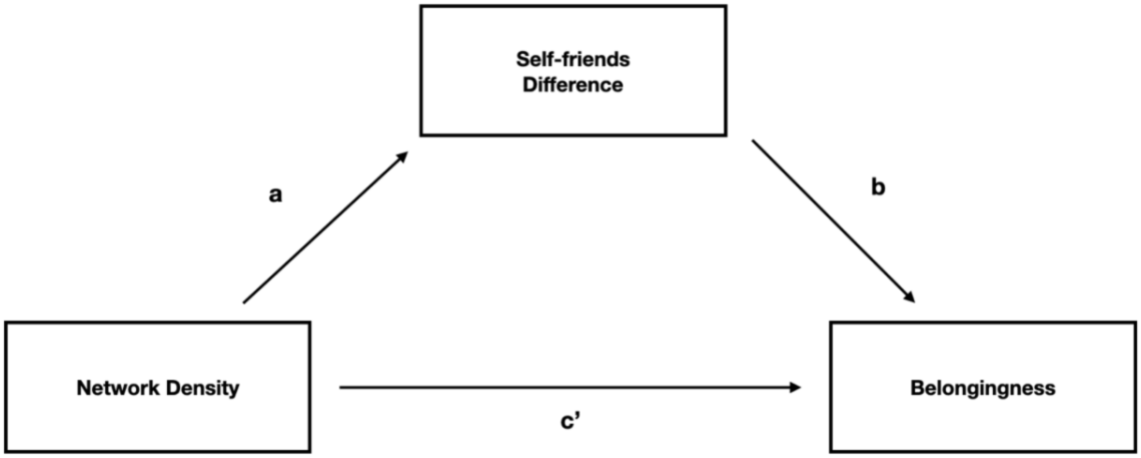

Type	a	b	cp	ab	total
Behaviors	-0.143***	-0.194***	0.16***	0.028***	0.188***
Values	0.048	-0.145***	0.195***	-0.007	0.188***

Figure 5. *Structure for each of the mediation models with self-friends difference as the mediator, with outputs in table. Highlighted is the significant indirect effect of self-friends behavioral difference that partially mediated the relationship between density and belongingness (***p<.001, **p<.01, *p<.05).*

Differences from friends partially mediate social identity effects on difference from average peer

People misperceive the average member of their wider community, partly because they are drawing on their closer networks to make such judgments. We therefore ask if self-friends differences mediate the relationship between social identity and self-average differences. We

specified exploratory mediation models for race, transfer, in-state, and first-generation student status (Figure 6) since these identity facets are related to belonging (Table 5). The negative indirect effect of race on *self-average behavioral difference* via *self-friends behavioral difference* was significant (b = .017, SE = .031, p = <.001, δ = .168, CI = [.107, .229]; a*b path in Figure 6). This pattern replicated for self-friends values difference mediating between race and self-average difference. We illustrate that differences in behavior from one's friends does lead to differences perceived from the "average student" for students who hold marginalized racial identities. This pattern for behavior differences reversed for first-generation and transfer students (Figure 6). We did not find *self-friends values differences* playing a mediating role between belonging and any other measured identities.

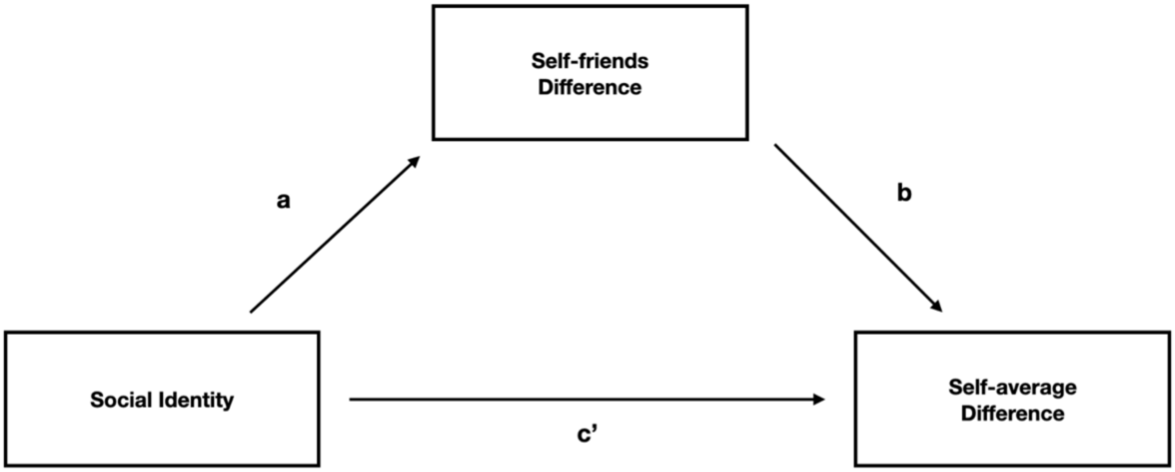

Identity	Type	a	b	cp	ab	total
Race	Behaviors	0.328***	0.512***	0.428***	0.168***	0.596***
	Values	0.119*	0.502***	-0.049	0.06*	0.011
Transfer	Behaviors	-0.547***	0.548***	0.027	-0.299***	-0.273*
	Values	-0.198	0.498***	-0.151	-0.099	-0.25*
First-Gen	Behaviors	-0.466***	0.52***	-0.49***	-0.242***	-0.732***
	Values	-0.057	0.499***	-0.131	-0.028	-0.16
In-state	Behaviors	-0.082	0.546***	-0.031	-0.045	-0.076
	Values	-0.099	0.496***	-0.194***	-0.049	-0.243***

Figure 6. *Structure for each of the mediation models with perceived difference from friends as the mediator, with outputs in table. Highlighted are the significant indirect effects of self-friends differences that partially mediated the relationship between social identity and self-average differences (***p<.001, **p<.01, *p<.05).*

Exploratory factor analysis to identify which types of behavior predict belongingness

To investigate which specific behaviors were most predictive of belongingness, we conducted an exploratory factor analysis on all 34 self-reported behaviors. Inspection of the scree plot (See OSF) indicated a four-factor solution was appropriate (Table 6). We calculated self-

average difference scores for each of the four resulting factors. We found that three of the four factor differences negatively predicted belonging (Table 7). These factors captured 1) use of university-specific language, 2) partying behavior, and 3) non-partying social behavior. The fourth factor captured cooking and grocery shopping – self-average differences in this domain were unrelated to belonging. This analysis suggests that belonging is affected by perceiving oneself to be different from peers on not just one, but on multiple facets of behavior—socializing, partying, and language use— these compound to create a general sense of belonging.

Behavior	F1	F2	F4	F3
Bodos	0.428835010	0.2555131704	0.13545442	0.073534311
Culbreth	0.509191075	0.1504697910	0.06563080	0.087948712
CornerNight	0.142582018	0.7007930850	0.16013778	0.023357795
Bridge	0.321817170	0.4387359855	0.08609320	-0.032080611
MadBowl	0.233425125	0.5855159248	0.05121553	-0.007538252
Football	0.300229112	0.3711512887	0.17601854	-0.051654955
Acapella	0.511540625	0.1102220595	0.10826754	-0.051040744
FoodTruck	0.316325836	0.1413521085	0.10068050	-0.265880443
Hike	0.583453479	0.0546074956	0.13505642	0.100902686
Picnic	0.619372961	0.0468812006	0.16693576	-0.013887655
Streak	0.463005375	0.1347624888	0.13032272	0.095782300
WeekOut	0.177823123	0.4966254599	0.02842375	0.089964689
TermGrounds	0.075857215	-0.0247202778	0.50383696	0.049716684
TermYear	0.050021508	0.0402483692	0.63939275	-0.025223385
AllNighter	0.452140651	0.1497562957	0.08681806	0.122774154
NOVAtalk	0.099952145	0.1097314877	0.39390747	-0.056603888
KhakisSkirts	0.275218997	0.3091174374	-0.05448710	0.065321532
DiningHall	0.005550375	-0.0163563682	0.07829788	-0.382528178
Exercise	0.089213495	0.1317877427	0.21527211	0.078570602
Cook	0.097307316	-0.0008915527	0.01677338	0.545829217
EatOut	0.049855204	0.2078127943	0.12541322	0.267685893
Grocery	0.173919083	-0.0101771612	0.12154335	0.569062352
TV	0.138193440	0.1050906268	0.08534511	0.216250311
Read	0.212634873	0.0503715513	0.06096515	0.109592759
Drive	0.087483689	0.0468527148	-0.02674320	0.459007341
Walk	0.067782246	0.0715557472	0.20952790	0.100482030
Study	0.009826688	0.0196267274	0.30302911	0.032326500
Clean	0.005096564	0.0672655767	0.17362486	-0.031850680
GoOutFriends	0.020236232	0.5000322599	0.24166201	0.013955036
Drink	0.141366671	0.6425264020	0.07880630	0.153117932
VideoGame	0.240467269	-0.0047711794	-0.17545916	0.079499883
SportOrg	0.368160391	0.1635797044	-0.09609486	0.087521225
SportRec	0.428352375	0.1388496062	-0.10930348	0.087756566
Family	0.195134928	0.1143256674	0.08731196	0.091132041

Table 6. *The four-factor solution for all behaviors.*

We used these factor loadings to compute self-average behavioral difference scores for each of the four domains. When we regress belonging on each of these self-average behavioral differences by factor, all but F3 significantly negatively predict belongingness. Greater self-average behavioral difference in using university-specific terms (b = -.068, SE = .016, t(1161) = -4.283, p < .001), partying (b = -.121, SE = .014, t(1161) = -8.788, p < .001), and non-partying social behavior (b = -.044, SE = .014, t(1161) = -3.08, p = .002), predicted lower levels of belonging. In other words, rating one's behavior very differently from the average across these domains separately predict belonging when controlling for one another (See Table 7 for model output). This pattern does not hold for F3 (cooking and grocery shopping). This analysis indicates that self-average differences in local behavior across domains matter for belonging to that locality.

	**belongingness**		
Predictors	*Estimates*	*CI*	*p*
(Intercept)	4.76	4.60 – 4.93	**<0.001**
F1	-0.04	-0.07 – -0.02	**0.002**
F2	-0.12	-0.15 – -0.09	**<0.001**
F3	0.01	-0.02 – 0.04	0.362
F4	-0.07	-0.10 – -0.04	**<0.001**
Observations	1166		
R^2 / R^2 adjusted	0.156 / 0.153		

Table 7. *Linear regression model output for self-average behavioral differences factors predicting belongingness.*

Behavior and value congruence predicts belonging

Here we present the complete model estimates for each polynomial multiple regression specified to examine congruence in either values or behaviors for self-average, self-friend, and average-friend comparisons (Table 8).

Variable	Dimension	IV1_Beta	IV2_Beta	IV1_Quad_Beta	IV2_Quad_Beta	Interaction_Beta
---	---	Self	Perceived average	Self	Perceived average	Self-average
Values	Self-transcendence	0.004	0.177***	0.034	-0.127***	0.078*
Values	Self-enhancement	0.102***	-0.093**	-0.046*	-0.046	0.125***
Values	Conservation	0.076*	0.034	-0.092***	-0.058*	0.144***
Values	Openness to change	0.062*	0.079**	-0.045	-0.074**	0.058*
Behaviors	Non-partying social behavior	0.311***	-0.192***	-0.052***	0.041*	0.037
Behaviors	Partying social behavior	0.419***	-0.168***	-0.045	-0.052**	0.012
Behaviors	Cooking	-0.015	0.059	0.023	-0.023	0.028
Behaviors	Language	0.268***	-0.185***	0.007	-0.055**	0.028
---	---	Self	Friends	Self	Friends	Self-friend
Values	Self-transcendence	0.013	0.029	0.018	-0.1***	0.116**
Values	Self-enhancement	0.074*	-0.001	-0.044	-0.057*	0.11**
Values	Conservation	0.089*	0.003	-0.081*	-0.076*	0.098*
Values	Openness to change	0.064	0.017	-0.048	-0.067**	0.059
Behaviors	Non-partying social behavior	0.314***	-0.017	-0.061**	-0.02	0.028
Behaviors	Partying social behavior	0.34***	0.027	-0.036	-0.107**	0.049
Behaviors	Cooking	-0.047	0.059	-0.009	-0.086	0.138**
Behaviors	Language	0.25***	-0.137*	-0.009	-0.101***	0.077*
---	---	Perceived average	Friends	Perceived average	Friends	Average-friend
Values	Self-transcendence	0.163***	-0.021	-0.163***	-0.103***	0.208***
Values	Self-enhancement	-0.102**	0.098**	-0.047	-0.047	0.102*
Values	Conservation	0.036	0.047	-0.073**	-0.107***	0.171***
Values	Openness to change	0.084*	0.01	-0.116***	-0.094***	0.167***
Behaviors	Non-partying social behavior	-0.226***	0.201***	0.024	-0.057***	0.029
Behaviors	Partying social behavior	-0.175***	0.357***	-0.037	-0.032	-0.023
Behaviors	Cooking	0.052	-0.014	-0.017	-0.003	0.014
Behaviors	Language	-0.129*	0.146**	-0.034	-0.03	0.019

Table 8. *Beta coefficients for each linear, quadratic, and interaction effects. Separate models compare either behavior factor or value dimension scores for either self-average, self-friend, or average-friend differences. Significant effects are indicated with stars (***p<.001, **p<.01, *p<.05).*

Friendship network density predicts (perceived) differences from the average student and friends

We predicted the density of students' social networks would impact their differences from the average student and their friends. To test this, we specify ten bivariate regressions with density predicting each difference score separately.

Density negatively predicted *self-average behavioral difference* (b = -.094, SE = .029, t(1161) = -3.267, p =.001 η^2 = .009, CI = [-.151, -.038]). Students with denser networks rated their behaviors closer to their ratings for an average university student. Density also negatively predicted *self-friends behavioral difference* (b = -.144, SE = .0281, t(1161) = -5.119, p <.001 η^2 = .022, CI = [-.199, -.089]); those with dense networks rated their behavior more similarly to their ratings for those friends. Lastly, density negatively predicted *average-friends values difference* (b = -.09, SE = .029, t(1161) = -3.118, p = .002 η^2 = .008, CI = [-.147, -.033]), meaning that students with more interconnected networks rated those friends' values closer to what they believed the average student valued. All other differences were unrelated to density (See OSF).

Behavioral differences predict total closeness to social network

We predicted that students would report lower levels of total closeness (the sum of the "closeness" scores they assigned to all their friends) if they had greater self-average differences. In other words, we expected students who feel dissimilar from their peers generally, or their friends specifically, would also report having fewer or less close friendships. To test this, we specified a model with all ten difference scores predicting total closeness (Figure 7). *Self-sample behavioral difference* negatively predicted total closeness (b = -.069, SE = .033, t(1170) = -2.105, p = .035, η^2 = .004, CI = [-.134, -.005]), such that students who rated their behavior farther from the sample mean behavior of their peers reported lower closeness with their friends. *Average-sample behavioral difference* positively predicts total closeness (b = .084, SE = .04, t(1170) = 2.124, p = .034, η^2 = .004, CI = [.006, .162]). Students who rated their friends' behaviors far from the sample mean behavior of their peers reported greater total closeness. As hypothesized, greater *self-friends behavioral difference* negatively predicted total closeness (b = -.15, SE = .37, t(1170) = -4.075, p < .001, η^2 = .014, CI = [-.222, -.078]). In other words, students who rated their behavior differently from their friends' reported lower levels of closeness with them. *Average-friends behavioral difference* negatively predicts closeness (b = -.108, SE = .041, t(1170) = -2.651, p = .008, η^2 = .006, CI = [-.188, -.028]); total closeness with friends decreases when students rate friends' behaviors differently from the average student. All other difference scores were insignificant in this model; behavioral differences matter most in predicting total closeness, when controlling for differences in values.

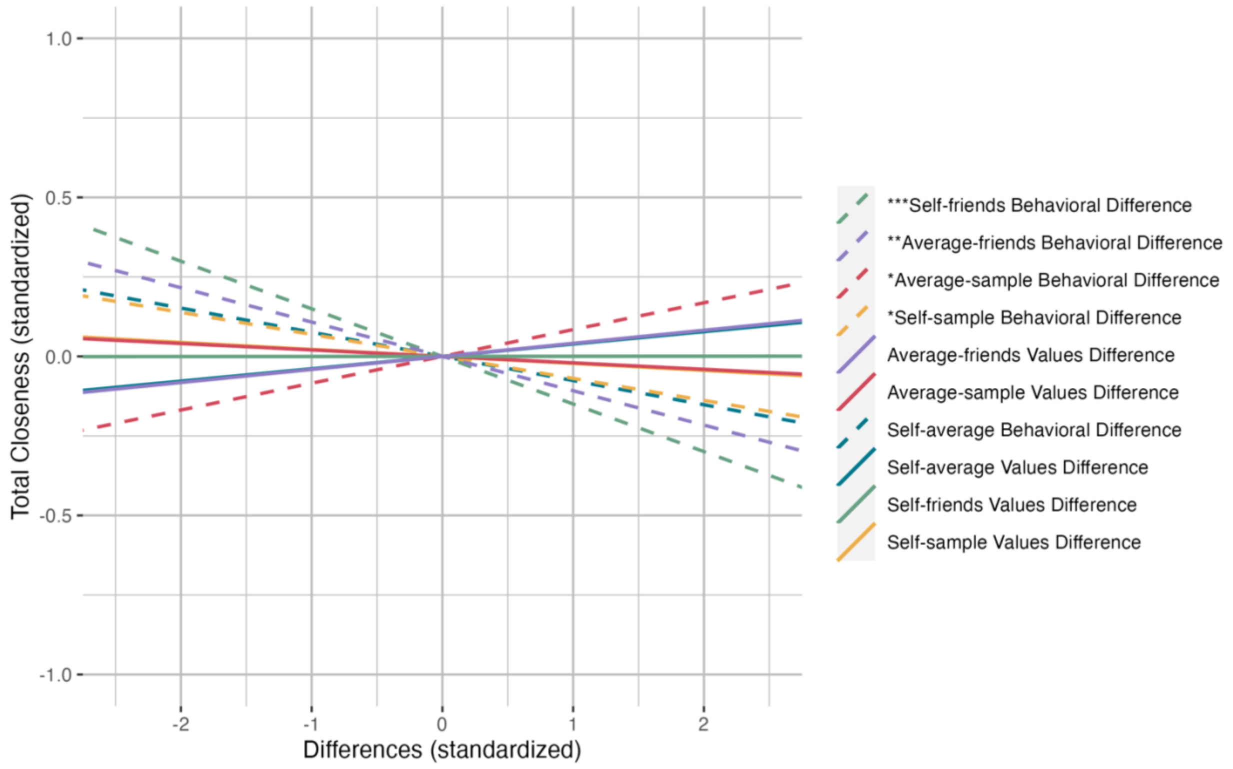

Figure 7. *Model predictions from a regression predicting total closeness using ten behavior and values differences scores (standardized). Dashed lines represent behavioral differences, while solid lines represent differences in values. (***p<.001, **p<.01, *p<.05).*

Self-friends differences predict total closeness

We additionally preregistered models examining the bivariate relationships between total closeness and perceived differences from those friends, in addition to the model reported in Figure 7. Total closeness negatively predicted *self-friends behavioral difference* (b = -.207, SE = .028, t(1179) = -7.268, p < .001) and *self-friends values difference* (b = -.065, SE = .029, t(1179) = -2.238, p = .025). In other words, students who reported greater total closeness to their friendship network reported their friends' behaviors or values closely to their own. These models replicate relationships reported in the main text.

Discussion of some supplementary materials results

The role of local versus general behavior. Perceived similarity in university-specific behaviors mattered more for belonging than perceived similarity on more generic behaviors. Perhaps people are more attuned to local behavior than daily habits that exist in any social context when considering how connected they feel to that locality. Indeed, architects theorize that consistent local behaviors and interaction with the environment can grant identity and belonging to that place (Sakhaeifar et al., 2016). An exploratory factor analysis of all self-reported behaviors (Supplementary Materials; Table 6) indicated that self-average differences in most of the specific behavioral domains separately predict belongingness; no particular subset of behaviors (such as partying) drives this effect (Supplementary Materials; Table 7). Future work should investigate if our effects rely on the tightness or looseness of permissible responses in the situations we asked people about (Gelfand et al., 2011). For example, there may be more variability in how often people have a picnic, rather than go to the dining hall; this could determine which behavioral norms are salient for belonging.

Total Closeness. In the present work, students who rated their behaviors closer to their perception of an average peer, their friends, and the sample mean had more close friends. This aligns with prior research showing that similarity (Floyd, 1995), and specifically perceived similarity, is linked to friendship intensity (Selfhout et al., 2009) and closeness (Secord & Backman, 1964). Our work is correlational, and future work should investigate if closeness causes (perceived) behavioral similarity between friends and community members or if perceiving yourself to be similar to your friends and community members increases your feelings of closeness, or your ability to forge close friendships. Researchers posit that behavioral similarity might stem from shared identity similarity generally, rather than explicit social contagion processes among close ties (Aral et al., 2009). Unfortunately, we only collected self-friends behavioral difference at one time point between a person and their entire network and we could not calculate differences from the sample mean of friends' behavior or values. Future work might measure similarity bidirectionally and between specific friends, rather than at the network level, to unpack these possibilities. Further, we collected egocentric social networks, so we cannot conclude how perceived similarity to friends or peers relates to one's position in the wider community. Future work might ask if perceived or actual similarity to the average student allows people to become social brokers, who connect otherwise disconnected groups (Burt, 1992).

Pilot Study

Methods

All study materials, data, and analysis scripts are openly available on OSF (https://osf.io/wv5r6/?view_only=56a582862beb4af89f5c6b4c4051828d).

Procedure. Participants (N = 1017) were recruited through the psychology participant pool at a large mid-Atlantic university in the United States for a 30-minute online Qualtrics study in Fall 2021. Participants were compensated with course credit.

Participants. Participants were 18 years of age or older, with an average age of 18.98. 64.19% of the sample identified as White, followed by 26.18% Asian American, then 7.85% Black / African American, 1.68% Other, and .10% Hawaiian Native & Pacific Islander. 62.45% of participants were in-state residents. 65.78% of the students identified as cis female, followed by 31.75% cis male, .99% non-binary, .89% other, .30% trans male, .20% gender fluid, and .10% trans female. Our sample was less racially diverse and had fewer participants identifying as male, compared to the greater university population (Supplementary Materials, Table 2).

Analytic Strategy. We conducted linear regression analyses in R (R Team, 2014). We conducted two linear regressions to study how all difference scores related to feeling different from the average and belongingness. To examine social identity variability in belongingness and difference scores, we conduct analyses of covariance using categorical variables for race, household income, current living location, in-state status, first-generation status, and transfer status as predictors. The social identity factors that were significant were tested via mediation models examining the relationships between social identity and belongingness via difference scores in behavior and values. For our mediation analyses, we utilized the sem() function from the lavaan R package (Rosseel et al., 2017). For complete analyses and anonymized data, see the OSF page. Results that did not replicate in the main text will be noted.

Results

Self-average difference in behavior and values predict feeling different. First, we asked whether our difference scores predict *feeling* different from the average student, by specifying a model with all difference scores predicting feeling different from the average student. This model provides a validity check for our self-average difference scores – do these indirect measures truly predict the subjective feeling of being different? *Self-average behavioral difference* (b = 1.873, SE = .918, t(968) = 2.04, p = .042) positively predicted feeling different from the average student. Students feel more different from the average when their behaviors were dissimilar to their own perception of an average peer's behavior (Figure 8). *Self-average values difference* (b = 3.633, SE = .98, t(968) = 3.709, p <.001) also positively predicted feeling different from the average; those who evaluated an average student's values very differently from their own felt more different from the average. Notably, *self-sample behavioral and values differences* between self-reports and the sample mean did not predict feeling different from the average. *Average-sample behavioral difference* positively predicted feeling different from the average (b = 2.462, SE = .863, t(968) = 2.853, p = .004), unlike in the main study. In other words, those with a greater mismatch between their perception of an average student's behavior and the sample mean felt more different from the average. Students who were more 'accurate' in their definition of an average student's behavior felt closer to the average. *Average-sample values difference* was unrelated to feeling different from the average peer. Self-average and average-sample differences were predictive of feeling different from the average when controlling for the true difference in both specific local behavior and more abstract values.

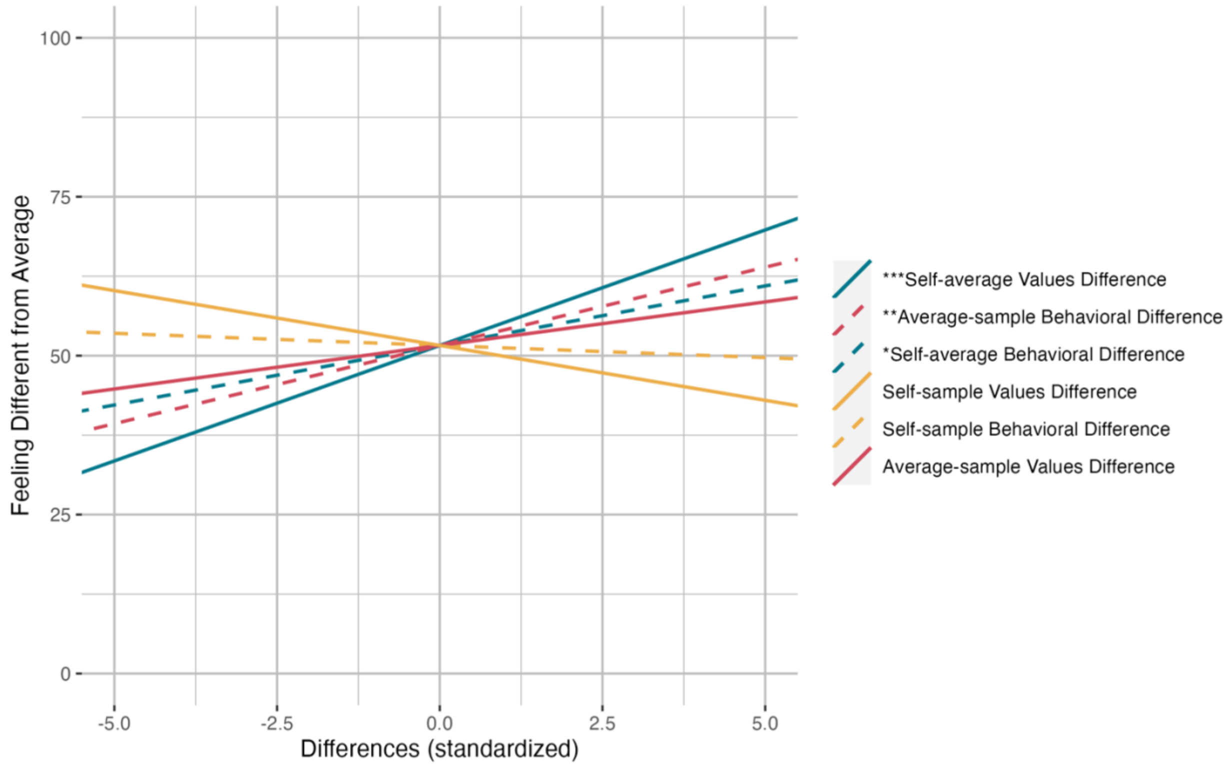

Figure 8. *Model predictions from a regression predicting participants' feelings that they are different from other students using all behavior and values differences scores (standardized). Dashed lines represent behavioral differences, while solid lines represent differences in values. (***p<.001, **p<.01, *p<.05)*

Self-average behavioral and value differences predict belongingness. We next specified a model with all difference scores predicting belongingness. *Self-average behavioral difference* negatively predicted belongingness (b = -.162, SE = .024, t(969) = -5.773, p < .001, η^2 = 0.033), meaning that those who rated a hypothetical average student's behavior as very different from their own reported lower levels of belongingness (Figure 9). *Self-average values difference* negatively predicted belongingness (b = -.173, SE = .03, t(969) = -5.703, p < .001, η^2 = 0.032); as with behavior, students who perceived the average student's values far from their own felt lower levels of belongingness. Surprisingly, *self-sample behavioral difference* positively predicted belongingness, when controlling for all other difference scores (b = .075, SE = .029, t(969) = 2.621, p = .009, η^2 = 0.007). In other words, students who rated their behavior differently from the sample mean reported significantly higher levels of belongingness, when controlling for the other difference scores. *Self-sample values differences* had no relationship with belongingness. *Average-sample behavioral difference* negatively predicted belongingness (b = -.061, SE = .027, t(969) = -2.289, p = .022, η^2 = 0.005), unlike in the main study. Students who perceived an average student's behavior differently from the sample mean reported lower levels of belonging. *Average-sample values difference* negatively predicted belongingness (b = -.073, SE = .035, t(969) = -2.107, p =.035, η^2 = 0.004), unlike in the main study; that is, students who rated the values of an average students very differently than the true mean had lower levels of belongingness. Generally, reduced feelings of belonging were associated with perceiving oneself to be different from the average student and being inaccurate about what the average student does and values.

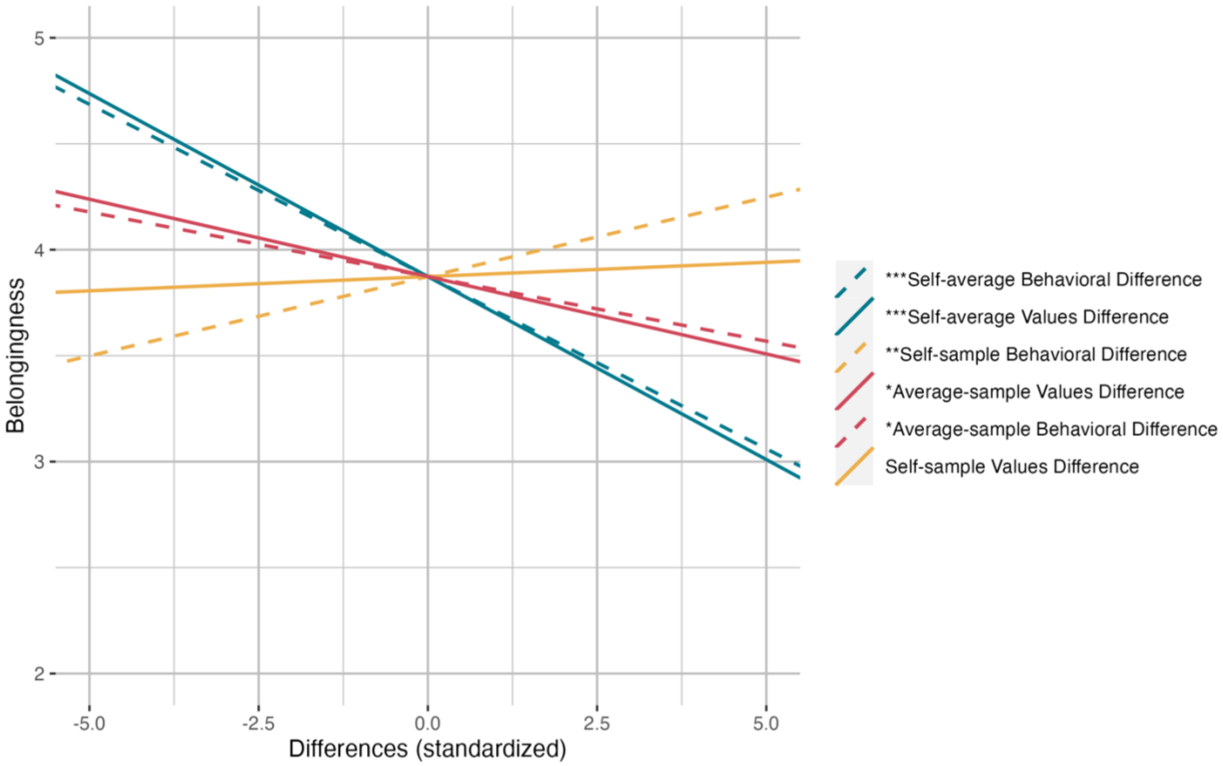

Figure 9. *Model predictions from a regression predicting belongingness using all behavior and values differences scores (standardized). Dashed lines represent behavioral differences, while solid lines represent differences in values. (***p<.001, **p<.01, *p<.05)*

Self-average differences mediate social identity impacts on belongingness. We know that behavior and value differences relate to belongingness (Figure 9), which led us to ask if these differences could mediate the relationship between social identity and belongingness. To avoid errors in assuming indirect effect significance without significant direct effects [52], we first investigated the relationships between social identity with belonging and with behavior and value differences.

We ran analyses of covariance (ANCOVA) examining social identity (race, household income, and in-state, first generation, and transfer student status, controlling for semesters spent on campus) predicting differences in levels of belongingness. We found a group effect of race ($F(1, 685) = 33.907$, $p < .001$), such that students of color reported lower levels of belongingness. Household income was significant in predicting belonging ($F(1, 685) = 16.115$, $p < .001$); students with household incomes below the state median reported lower belongingness. We found that in-state ($F(1, 685) = 14.295$, $p < .001$), and transfer student status ($F(1, 685) = 6.806$, $p = .009$) displayed group differences in belongingness, such that out-of-state and transfer students reported lower belonging (See OSF).

In separate ANCOVAs, we found identity variability in some of the difference scores (See OSF for model outputs); we therefore tested for mediation with race, income, in-state status, and transfer student status.

Given that both difference scores and social identity predict belongingness, we asked if these differences in behavior and values mediate marginalized students' feelings of belongingness. We conducted separate mediation models for each social identify variable that significantly predicted belonging, using the difference scores as simultaneous mediating

variables. We specified structural equation models with a) a direct path from social identity to belongingness, b) indirect paths from social identity to belongingness via self-sample, self-average, and average-sample behavioral or value differences in separate models, and c) covariances between difference scores (Figure 10; See OSF for details). We also estimated the statistical significance of d) the three indirect effects, e) the total effect (direct and indirect effects combined) and f) the contrasts between the three indirect effects, which indicate whether one mediator is a significantly stronger mediator than another.

We first examined if behavioral differences would mediate the relationship between race and belongingness. The indirect effect of race (with non-White coded as 0.5 and non-Hispanic White coded as -0.5) on belongingness via *self-average behavioral difference* (a2*b2 path in Figure 10) was significant (b = -.037, SE = .0125, p = .003, CI = [-.062, -.013]), while controlling for self-sample and average-sample behavioral differences. The contrast comparing self-average behavioral differences to self-sample (b = -.037, SE = .014, p = .005, CI = [-.066, -.01]) and average-sample behavioral differences (b = -.038, SE = .014, p = .00`8, CI = [-.066, -.01]) was significant. Self-average behavioral differences matter, over and above self-sample or average-sample behavioral differences, for belongingness of students of color.

This pattern replicates for household income, in-state residence, and transfer student status (Figure 10), such that students identifying with the minority group have greater *self-average behavioral differences* and thus lower levels of belongingness. Furthermore, *self-average values difference* partially mediates the relationships between household income and in-state status on belongingness while controlling for self-sample and average-sample differences, unlike in the main study (Figure 10). Self-average values differences did not partially mediate the relationship between social identity and belongingness for models testing racial identity or transfer student status.

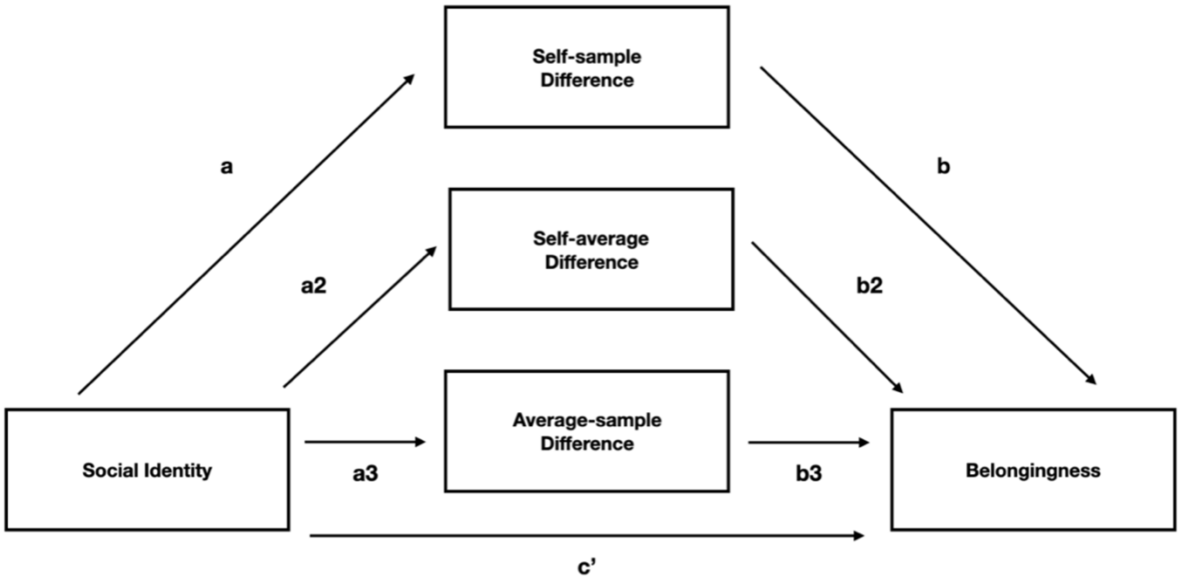

Identity	Type	a	a2	a3	b	b2	b3	cp	ab	a2b2	a3b3	total
Race	Behaviors	0.021	0.239***	-0.008	0.038	-0.156***	-0.072**	-0.488***	0.001	-0.037**	0.001	-0.524***
	Values	0.06	0.075	0.061	0.01	-0.186***	-0.071*	-0.506***	0.001	-0.014	-0.004	-0.524***
Income	Behaviors	0.42*	0.925**	0.284	0.002	-0.063***	-0.021	-0.477***	0.001	-0.059**	-0.006	-0.541***
	Values	0.218*	0.302**	0.15	0.005	-0.195***	-0.04	-0.477***	0.001	-0.059**	-0.006	-0.541***
In-state	Behaviors	-0.073	-0.419*	-0.062	0.004	-0.059***	-0.037*	0.252***	0	0.025*	0.002	0.278***
	Values	-0.038	-0.137*	-0.033	0.007	-0.181***	-0.069*	0.252***	0	0.025*	0.002	0.278***
Transfer	Behaviors	0.96	4.592*	0.677	0.004*	-0.011***	-0.006**	-0.265**	0.004	-0.048*	-0.004	-0.314***
	Values	-0.089	-0.094	0.111	0.01	-0.185***	-0.074*	0.305***	-0.001	0.017	-0.008	0.314***

Figure 10. *Structure for each of the mediation models, with outputs in table. Highlighted are the significant indirect effects of self-average differences (whether behavior or values; a2b2 path) that partially mediated the relationship between social identity and belongingness when controlling for self-sample and sample-average differences (***p<.001, **p<.01, *p<.05).*

Sample representativeness of pilot study

The sample is representative of the 2020 undergraduate population at the university, except for racial and gender identities. The sample is racially imbalanced when compared to the overall undergraduate student population.

**Race**	**Population** (*Undergraduate Students…*, 2020).	**Sample**
White non-Hispanic	55.67%	64.19%
Asian	16.16%	26.18%
African-American / Black	6.74%	7.85%
Hispanic / Latinx	6.73%	
Multi-racial	5.16%	

Unknown	5.13%	1.68%
Non-resident alien	4.24%	
Native American / Alaskan	.08%	
Native Hawaiian / Pacific Islander	.08%	.1%

Table 9. *Racial demographics of sample and population.*

Table 9 reflects an overrepresentation of the three main racial groups in the sample: White, Asian, and Black / African American, meaning there was less racial diversity in the sample than in the population overall. The undergraduate student population is 56% female and 44% male (*College Navigator…*, 2022), but in our sample 65.78% of the students identified as cis female, followed by 31.75% cis male, .99% non-binary, .89% other, .30% trans male, .20% gender fluid, and .10% trans female. There were fewer male-identifying students in our sample.

Pilot Study: Difference Score Descriptive Statistics

Figure 11 shows the histograms for the six difference scores in the pilot study, before they were centered and standardized for analyses.

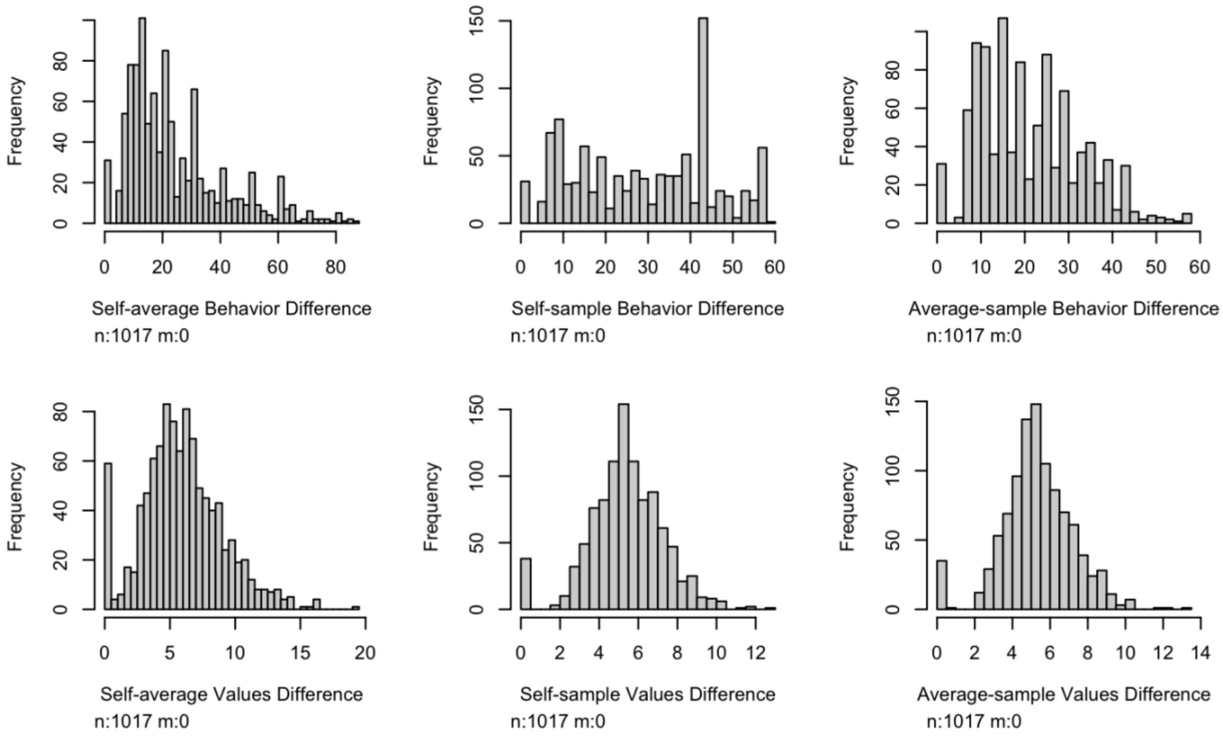

Figure 11. *Histograms of the raw difference scores.*

Pilot Study: Differences in behavior and values predicting belongingness via bivariate correlations

When we conduct bivariate correlations, as difference from the average student increases, belongingness decreases. However, in the full model, self-sample behavioral and value

differences predicted *greater* levels of belongingness, likely due to a suppression effect by the other covariates. See Figure 12 for the bivariate correlations.

Although two difference scores, self-sample and average-sample behavioral difference, were insignificantly related to belongingness, we still include them in the full model within our main manuscript. It is inappropriate to conduct bivariable selection to include covariates in a model for many reasons (Sun et al., 1996).

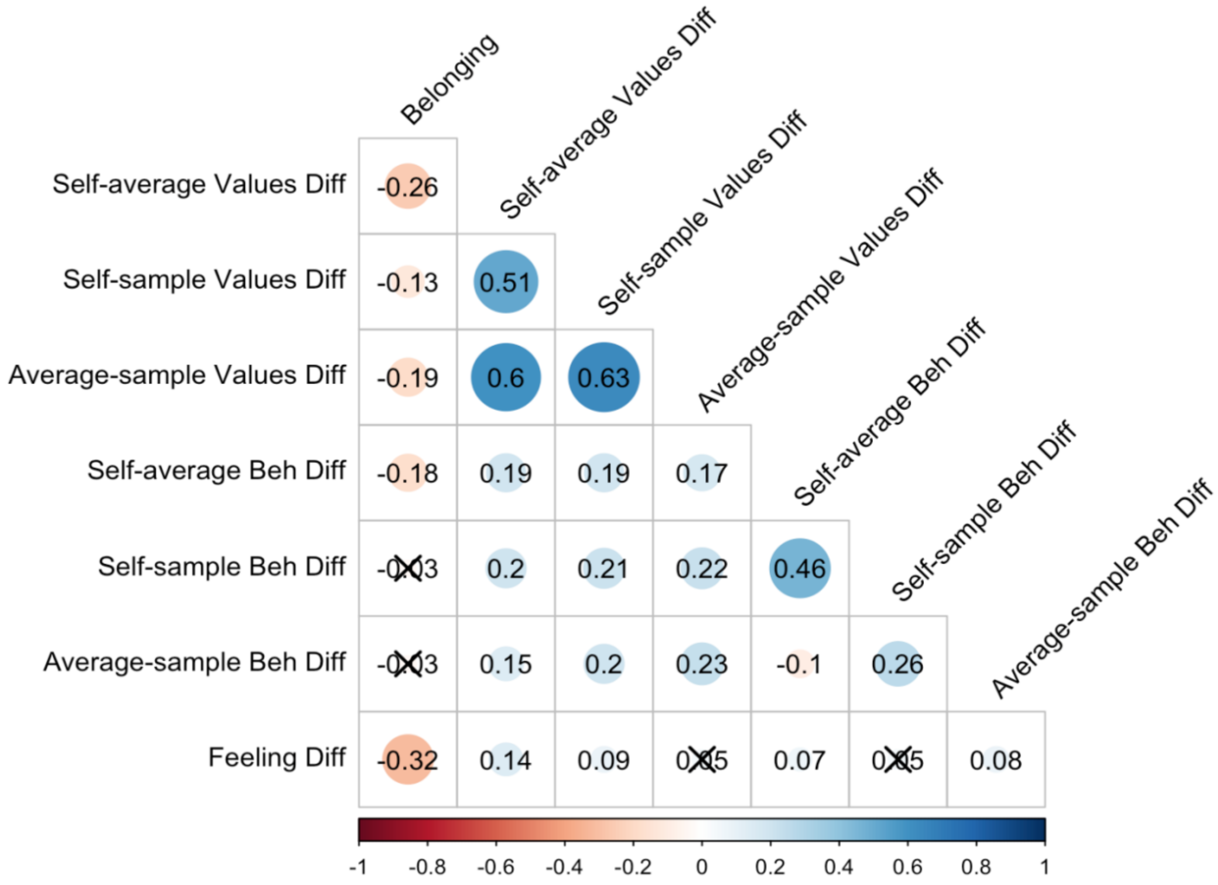

Figure 12. *Correlation matrix indicating significant bivariate relationships of key variables.*

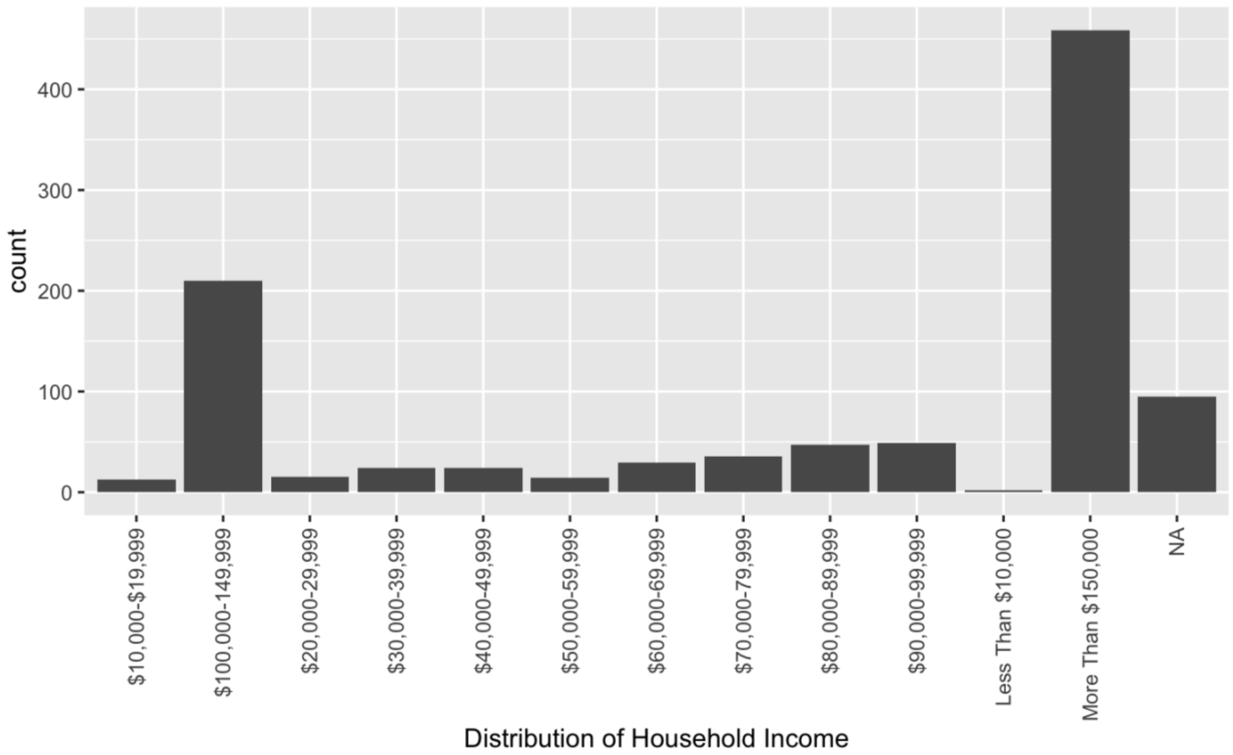

Figure 13. *Histogram of household income for pilot study sample.*
